# Genomic Prediction and Association Mapping of Curd-Related Traits in Gene Bank Accessions of Cauliflower

**DOI:** 10.1534/g3.117.300199

**Published:** 2017-12-18

**Authors:** Patrick Thorwarth, Eltohamy A. A. Yousef, Karl J. Schmid

**Affiliations:** * Institute of Plant Breeding, Seed Science and Population Genetics, University of Hohenheim, 70599 Stuttgart, Germany; † Department of Horticulture, Faculty of Agriculture, University of Suez Canal, Ismailia 41522, Egypt

**Keywords:** cauliflower, gene bank, genome-wide association study, genomic prediction, genotyping-by-sequencing, GenPred, Shared Data Resources, Genomic Selection

## Abstract

Genetic resources are an important source of genetic variation for plant breeding. Genome-wide association studies (GWAS) and genomic prediction greatly facilitate the analysis and utilization of useful genetic diversity for improving complex phenotypic traits in crop plants. We explored the potential of GWAS and genomic prediction for improving curd-related traits in cauliflower (*Brassica oleracea* var. *botrytis*) by combining 174 randomly selected cauliflower gene bank accessions from two different gene banks. The collection was genotyped with genotyping-by-sequencing (GBS) and phenotyped for six curd-related traits at two locations and three growing seasons. A GWAS analysis based on 120,693 single-nucleotide polymorphisms identified a total of 24 significant associations for curd-related traits. The potential for genomic prediction was assessed with a genomic best linear unbiased prediction model and BayesB. Prediction abilities ranged from 0.10 to 0.66 for different traits and did not differ between prediction methods. Imputation of missing genotypes only slightly improved prediction ability. Our results demonstrate that GWAS and genomic prediction in combination with GBS and phenotyping of highly heritable traits can be used to identify useful quantitative trait loci and genotypes among genetically diverse gene bank material for subsequent utilization as genetic resources in cauliflower breeding.

Wild ancestors, landraces, old varieties, and breeding stocks of crop plants that are preserved in *ex situ* gene banks are an important resource of genetic diversity for introducing favorable alleles into modern cultivars. Adaptation to unfavorable environmental conditions, disease resistance, and nutritional value are traits for which new alleles can be found in genetic resources (Hawkes 1991). The improvement of quantitative traits such as yield with genetic resources is more challenging because of deleterious alleles and missing adaptation to modern agricultural practices. However, new improvement strategies may be used for such traits (Longin and Reif 2014). Molecular breeding methods such as association mapping and genomic selection combined with current sequencing technology offer new possibilities for an efficient utilization of genetic resources (Tester and Langridge 2010; Bevan *et al.* 2017).

Cauliflower (*Brassica oleracea* var. *botrytis*, 2*n* = 2× = 18) is an important vegetable crop, owing to its high nutritional value (Picchi *et al.* 2012). With an annual production area of ∼1.4 million ha and 24 million tons harvested in 2014 (http://faostat.fao.org/), cauliflower is of great economic importance, particularly in Asia. The most important trait and yield determinant is the cauliflower curd (edible, often white inflorescence meristem; Li *et al.* 2017), but response to vernalization is also an important agronomic trait, and genotypes with obligate and facultative vernalization requirements have been identified (Matschegewski *et al.* 2015).

Genome-wide association studies (GWAS) have been carried out in major cereal crops, including maize (Li *et al.* 2012), rice (Norton *et al.* 2014), barley (Gawenda *et al.* 2015), and wheat (Edae *et al.* 2014). In *Brassicaceae*, GWAS have mainly been conducted in rapeseed (*Brassica napus*) to dissect the genetic basis of disease resistance (Jestin *et al.* 2011), seed oil content and quality (Zou *et al.* 2010; Rezaeizad *et al.* 2011), seed weight and quality (Li *et al.* 2014), seed glucosinolate content (Hasan *et al.* 2008), and several morphological and phenological traits (Cai *et al.* 2014). Recently, GWAS was used in cauliflower to identify flowering-related quantitative trait loci (QTL) (Matschegewski *et al.* 2015), which are useful for breeding varieties adapted to different temperature regimes. For example, expression analysis identified a homolog of the *Arabidopsis thaliana CDAG1* (*Curd Development Associated Gene 1*) gene in cauliflower, whose overexpression increased curd yield (Li *et al.* 2017).

In addition to genetic mapping, genomic selection (Meuwissen *et al.* 2001) has become an important method in plant breeding (Schmid and Thorwarth 2014). Genomic selection is based on the prediction of breeding values based on marker information alone. A training population with genotypic and phenotypic information is used to estimate marker effects with a statistical model, which is then applied to calculate the breeding values of the potential selection candidates in the breeding population (Heffner *et al.* 2009). Cross-validation allows selection of the best model for a certain population, trait, and genetic architecture (Crossa *et al.* 2010). Genomic selection provides several benefits in both animal and plant breeding (Hayes *et al.* 2009; Heffner *et al.* 2009; Jannink *et al.* 2010), including more rapid breeding cycles and fewer field trials, leading to increased genetic gain per time unit at a lower cost (Schaeffer 2006; König *et al.* 2009; Heffner *et al.* 2010). Genomic prediction has been successfully applied in wheat (Poland *et al.* 2012b), maize (Crossa *et al.* 2013), and soybean breeding (Jarquín *et al.* 2014). In *Brassicaceae*, one study investigated the potential of genomic selection in rapeseed (Würschum *et al.* 2014) and concluded that it is a promising method for rapeseed breeding.

Advances in sequencing technology allow the detection of single-nucleotide polymorphisms (SNPs) from large and diverse germplasm collections (Crossa *et al.* 2013). Genotyping-by-sequencing (GBS) generates tens of thousands of molecular markers at low cost (Elshire *et al.* 2011; Poland *et al.* 2012b; Sonah *et al.* 2013) and is an efficient tool for plant genetics and breeding (Poland and Rife 2012; Fu *et al.* 2014; Tardivel *et al.* 2014). It has been successfully used for genomic selection in wheat, maize, and soybean (Poland *et al.* 2012a; Crossa *et al.* 2013; Jarquín *et al.* 2014), and in GWAS of morphological traits and flavonoid pigmentation in maize and sorghum, respectively (Romay *et al.* 2013; Morris *et al.* 2013). Recently, Zhao *et al.* (2016) used specific-locus amplified fragment sequencing to create a genetic map of cauliflower. One disadvantage of GBS is a high proportion of missing data, which may be alleviated by genotype imputation (Poland and Rife 2012), although the value of imputation for association mapping and genomic prediction is debated (Marchini and Howie 2010; Rutkoski *et al.* 2013).

In this study, we combined GBS with a phenotypic analysis of cauliflower genetic resources to investigate the potential of GWAS for the identification and genomic prediction for the selection of useful genetic variation in cauliflower breeding. More specifically, the main objectives of this study were (1) to identify SNP markers that are associated with phenotypic variation in curd-related traits using GWAS, (2) to quantify the predictive ability of genomic prediction models for curd-related traits in diverse cauliflower genotypes obtained from *ex situ* gene banks, and (3) to evaluate the effect of data imputation on association mapping and genomic prediction. We show that GWAS identifies genomic regions harboring potentially useful variation and that genetic resources are suitable for genomic prediction of phenotypic variation in highly heritable traits.

## Materials and Methods

### Plant materials and phenotyping

A total of 192 cauliflower accessions representing a wide range of morphological diversity and geographical origins were obtained from the gene banks of the United States Department of Agriculture (USDA) and from the Leibniz-Institut für Pflanzengenetik und Kulturpflanzenforschung (IPK) Gatersleben, Germany. Detailed information about accessions is given in Supplemental Material, Table S1 in File S1. All accessions were phenotyped for curd-related traits in replicated field trials at two locations (experimental stations: Heidfeldhof and Kleinhohenheim in Stuttgart, Germany), for three successive growing seasons (June 2011, April 2012, and August 2012). The field experiment was conducted in randomized complete block design with two replications (Yousef *et al.* 2015). Five ripened curds were harvested from each plot and used to measure six traits that reflect various aspects of curd development and morphology according to Lan and Paterson (2000).

1. Curd width (cm): width of the curd.
2. Cluster width (cm): width of the largest floral cluster.
3. Number of branches: number of branches within the curd that originated from the main stem.
4. Apical shoot length (cm): stem length from the apical meristem to where the closest first-rank branch originated from the main stem.
5. Nearest branch length (cm): length of the branch that is nearest to the apical meristem.
6. Days to budding: number of days from planting to appearance of the first floral bud.

### Genotyping and marker imputation

We genotyped the material with the original GBS protocol using the *ApekI* restriction enzyme (Elshire *et al.* 2011). The sequencing and bioinformatic analyses of our material are described in Yousef *et al.* (2017). Briefly, 192 genotypes were sequenced as two sequencing libraries of 96 individuals on an Illumina HiSeq 1000, using a set of in-house scripts and public sequence analysis tools including bwa (Li and Durbin 2009) and FastQC (http://www.bioinformatics.babraham.ac.uk/projects/fastqc/). Eighteen genotypes with <300,000 reads were excluded from further analyses, which resulted in a total sample of 174 accessions for further analyses. The preprocessed reads were aligned to the genome of *B. oleracea* sp. *capitata* (Liu *et al.* 2014) using bwa. SNP calling was performed with SAMtools (Li and Durbin 2009), bcfutils, vcfutils, and custom Python scripts. The vcf file was parsed to retain only SNP positions with a coverage of ≥30, and ≥10 reads confirming the variant nucleotide. In the end, 120,693 SNPs were detected with 19.02–76.73% of missing values per genotype (File S2).

We imputed missing genotypes with fastPHASE (Scheet and Stephens 2006) and BEAGLE (Browning and Browning 2007); both use a hidden Markov model to cluster haplotypes but they differ in the underlying model. The fastPHASE method uses an expectation–maximization algorithm for parameter estimation and fixes the number of haplotype clusters in the model. By contrast, BEAGLE uses empirical frequencies and allows the cluster number to be changed at each locus for a better fit to the localized linkage disequilibrium (LD) (Pei *et al.* 2008). We used fastPHASE as the main imputation method because we expected it to perform better than BEAGLE with our data set, which is characterized by a low LD, small sample size, and high marker density. The advantages of imputation methods under different scenarios were described by Browning and Browning (2011). We included BEAGLE for comparison for some analyses, but used only fastPHASE-imputed data for the GWAS to keep the number of analyses manageable. SNP markers with minor allele frequency (MAF) <0.05 and any missing values were excluded from further analyses, which resulted in a total of 675 markers (unimputed data set). Next, SNP alleles were imputed with fastPHASE (Scheet and Stephens 2006) and markers with a MAF <0.05 were excluded, which resulted in a total of 64,372 SNPs (imputed data set with fastPHASE). Genomic prediction was conducted with a third data set, in which SNP alleles were imputed with BEAGLE 4 (Browning and Browning 2007) and markers with a MAF <0.05 were excluded, which resulted in a total of 62,566 SNPs (imputed data set with BEAGLE).

### Analysis of phenotypic variation

The effects of the genotype and environment (environment was treated as the combination of location and season) and their interactions with phenotypic variation were evaluated using analysis of variance with the aov function of R (R Core Team 2015). Details of the phenotypic data analysis are described in Yousef *et al.* (2015). A mixed-effects model was fitted using restricted maximum likelihood (REML) with the lmer function from the R package lme4 (version 1.0-5) (Bates *et al.* 2014):

$$y_{ij}=\mu +G_{i}+E_{j}+GE_{ij}+e_{ij},$$ (1)

where $y_{ij}$ are the adjusted means of the $i^{th}$ genotype in the $j^{th}$ environment, *m* is the overall mean, $G_{i}$ is the effect of the $i^{th}$ genotype, $E_{j}$ is the effect of the $j^{th}$ environment, and $GE_{ij}$ the genotype × environment interaction. All effects were considered as random, except the intercept, which was treated as a fixed effect. Variance components of this model were used to calculate broad sense heritability for each trait according to Nyquist and Baker (1991) as

$$H^{2}=\frac{\sigma_{g}^{2}}{\sigma_{g}^{2}+\frac{\sigma_{ge}^{2}}{e}+\frac{\sigma_{e}^{2}}{re}},$$ (2)

where $H^{2}$ is the broad sense heritability, $\sigma_{g}^{2}$ is the genetic variance, $\sigma_{ge}^{2}$ is the genotype-by-environment variance, $\sigma_{e}^{2}$ is the error variance, *r* is the number of replications, and *e* is the number of environments. Best linear unbiased predictors (BLUPs) for the genotypic effects were extracted from model (1) and used to calculate the genetic correlation ($r_{G}$) among all traits. The genetic and phenotypic correlation coefficients are based on the Pearson correlation coefficient.

### Population structure and LD

We used a discriminant analysis of principle components (DAPC) to infer clusters of genetically related individuals by using a k-means algorithm as implemented by Jombart and Ahmed (2011). LD between adjacent markers was calculated and the LD decay over distance for each chromosome was assessed. To identify differences in LD levels between the complete sample and the clusters identified by k-means, we used PLINK (Purcell *et al.* 2007) to calculate LD as:

$$r^{2}=\frac{{\left(p_{ab}-p_{a}p_{b}\right)}^{2}}{p_{a}\left(1-p_{a}\right)p_{b}\left(1-p_{b}\right)},$$ (3)

where $p_{ab}$ is the frequency of haplotypes with allele *a* at one locus and allele *b* at the other locus (VanLiere and Rosenberg 2008). The extent of background LD was estimated as the correlation of the 95% percentiles of all pairwise markers between chromosomes (Breseghello and Sorrells 2006). Additionally, we analyzed the persistence of linkage phase between DAPC-inferred clusters and the whole sample to validate whether a marker effect estimated in one cluster will contribute to the prediction ability in other clusters. Persistence of linkage phase is calculated as correlation coefficient *r* by:

$$r=\frac{D}{\sqrt{p_{a}\left(1-p_{a}\right)p_{b}\left(1-p_{b}\right)}}$$ (4)

where $D=p_{ab}-p_{a}p_{b}.$ As a measure of LD, *r* ranges from −1 to 1 (De Roos *et al.* 2008). Persistence of linkage phase is expressed as correlation of *r* between the same chromosomes of each cluster. The number of markers differed between clusters; therefore, we averaged the correlation of *r* values between clusters over groups of 50 markers.

### Association mapping

GWAS was performed with the R (R Core Team 2015) implementations of the Efficient Mixed Model Association eXpedited (EMMAX; Kang *et al.* 2010) and the Multi Locus Mixed Model (MLMM Segura *et al.* 2012) methods. The MLMM analysis was conducted with R scripts available at https://github.com/Gregor-Mendel-Institute/mlmm. EMMAX is a method that uses a linear model in combination with a marker-derived relationship matrix to correct for population structure. The linear model has the form:

y=Xβ+Zu+e, (5)

where y is the vector of phenotypes; X is a matrix containing the markers; β is the vector of fixed-effects coefficients; Z is an incidence matrix; u is the random effect, where $Var\left(u\right)=\sigma_{a}^{2}K$, with K representing the relationship between genotypes inferred from genetic markers; and e is the residual effect with $e\;∼\;N(0,\;\sigma_{e}^{2}I)$. Additive genetic variance ($\sigma_{a}^{2}$) and environmental variance ($\sigma_{e}^{2}$) are derived from the REML estimates. Variance components are only calculated once and are taken as fixed in EMMAX, which speeds up computation (Kang *et al.* 2010). MLMM uses the same linear model as EMMAX, but additionally includes significant SNPs as covariates in the model by using a forward–backward stepwise algorithm with reestimation of variance parameters ($\sigma_{a}^{2}$ and $\sigma_{e}^{2}$) at each step.

Following Utz *et al.* (2000), the proportion of phenotypic variance explained by a QTL was calculated as:

$$R_{adj}^{2}=R^{2}-\;\left(\frac{z}{N-z-1}\right)\left(1-R^{2}\right),\;$$ (6)

where $R^{2}$ is the coefficient of determination, *z* is the number of predictors (number of significant SNPs in GWAS), and *N* is the number of observations. The proportion of genotypic variance explained was calculated as:

$$\hat{\rho }=\frac{R_{adj}^{2}}{{\hat{H}}^{2}},\;$$ (7)

where ${\hat{H}}^{2}$ is the heritability of a given trait as defined in equation (2).

Confounding effects due to population structure were evaluated with the inflation factor λ, which is the ratio of the observed median to the expected median of a test statistic distribution (Devlin and Roeder 1999). Values close to 1 indicate no inflation. The significance threshold was set for the unimputed and imputed data for each trait separately using the false discovery rate (FDR; Benjamini and Hochberg 1995), where FDR values are computed from the *P*-values. The FDR was set to 0.2 for all data sets.

### Genomic prediction

We evaluated three different genomic prediction methods, namely genomic BLUP (GBLUP), ridge regression BLUP (RRBLUP), and BayesB. The GBLUP model uses a realized relationship calculated from genetic markers (Habier *et al.* 2013) and is defined as:

y=1μ+Zg+e, (8)

where g is an *n* × 1 vector of random effects and Z is the design matrix. Genetic values are modeled as random effects with $g∼N\left(0,G\sigma_{g}^{2}\right),$ with $\sigma_{g}$ as the genetic variance and G the realized relationship matrix.

An RRBLUP model (Meuwissen *et al.* 2001) was used to estimate the marker effects and to calculate the prediction ability. The model is of the form:

y=1μ+Xβ+e, (9)

where y is the vector of *n* phenotypic records, μ is a vector of fixed effects that represents the overall mean, β is an *n* × 1 vector of random effects, and X is the marker matrix. The residuals follow a normal distribution $e∼N\left(0,I\sigma^{2}\right),$ where I is the identity matrix. The GBLUP and RRBLUP implementations of the *synbreed* R package (Wimmer *et al.* 2012) were used.

Additionally, a BayesB (Meuwissen *et al.* 2001) model as implemented in the BGLR R package (Pérez and de los Campos 2014) was used to estimate marker effects and to calculate prediction ability. BayesB uses the same linear model as RRBLUP, but a prior for the marker effects is modeled as mixture of a point of mass at zero and a slab that has a scaled-t density. Following Pérez and de los Campos (2014), the notation of the prior distribution is:

$$p\left(\beta_{j},\sigma_{\beta }^{2},\pi \right)=\left\{\prod_{k}\left[\pi N\left(\beta_{jk}\left|0,\sigma_{\beta }^{2}\right.\right)+\left(1-\pi \right)1\left(\beta_{jk}=0\right)\right]\chi^{-2}\left(\sigma_{\beta_{jk}}^{2}\left|df_{\beta }\right.,S_{\beta }\right)\right\}B\left(\pi \left|p_{0},\pi_{0}\right.\right)\times G(S_{\beta }\left|r,s\right.),$$ (10)

where β represents the vector of regression coefficients and $\sigma_{\beta }^{2}\;$is the respective variance. Parameter π is the proportion of nonzero effects and follows a β prior distribution, which implies the possibility of variable selection. $N\left(\beta_{jk}\left|0,\sigma_{\beta }^{2}\right.\right)$, $\chi^{-2}\left(\sigma_{\beta_{jk}}^{2}\left|df_{\beta }\right.,S_{\beta }\right)$, $B\left(\pi \left|p_{0},\pi_{0}\right.\right)$, and $G(S_{\beta }\left|r,s\right.)$represent the normal, chi-squared, β, and γ density distributions, respectively (Pérez and de los Campos 2014). BayesB was chosen over other Bayesian models such as BayesA and BayesC because it assumes an *a priori* distribution of marker effects following a mixture distribution with point mass at zero and a scaled-t slab similar to BayesA, and utilizes both shrinkage and variable selection, similar to BayesC (Pérez and de los Campos 2014). The hyper-parameters were chosen according to the default values in BGLR.

Since population structure can inflate prediction ability (Guo *et al.* 2014), we estimated the effect of population structure on prediction ability by directly correcting the kinship matrix in the GBLUP model for confounding effects of population structure (Thorwarth *et al.* 2017). The corrected kinship matrix was calculated with PC-Relate (Conomos *et al.* 2016). Prediction ability was defined as correlation between observed phenotypic and predicted genotypic values [$cor(y,\;\hat{g})$] and was calculated by a five-fold cross-validation with 10 replications for each trait. To test whether genotypic effects in the genomic prediction and GWAS were caused by the same QTL, the position of SNPs with the greatest effects in genomic prediction models were compared with the most significant SNPs in the GWAS.

### Data availability

Raw sequence data have been submitted to the Sequence Read Archive under accession number PRJEB8701. SNP calls are available on figshare at https://doi.org/10.6084/m9.figshare.5709784. Best Linear Unbiased Estimators (BLUEs) of phenotypic data are provided as supplementary material (File S2).

## Results

### Phenotypic analysis of the six yield-related traits

The 174 gene bank accessions were evaluated for six curd-related traits and exhibited a large phenotypic variation in all traits (Yousef *et al.* 2015). For example, number of days to budding ranged from 45 to 118 d with an average of 75.24±11.68 d, and curd width ranged from 11.11 to 15.29 cm with an average of 13.52±0.73 cm (Table S2 in File S1). Analysis of variance showed that all traits were strongly affected by genotype (G), environment (E) and genotype by environment interaction (G × E; P<0.001). Broad-sense heritability ($H^{2}$) differed strongly between traits. The traits of cluster width and number of days to budding showed moderate (56%) and high (94%) heritabilities, respectively. Furthermore, the traits of curd width and cluster width showed high phenotypic ($r_{p}=0.69$) and genotypic ($r_{g}=0.59$) correlations (Table S3 in File S1), as did apical length and nearest branch length ($r_{p}=0.79$ and $r_{g}=0.71$). Number of days to budding was negatively correlated with number of branches ($r_{p}=-0.22$ and $r_{g}=-0.23$).

### Analyses of population structure and LD decay

**Figure 1:**
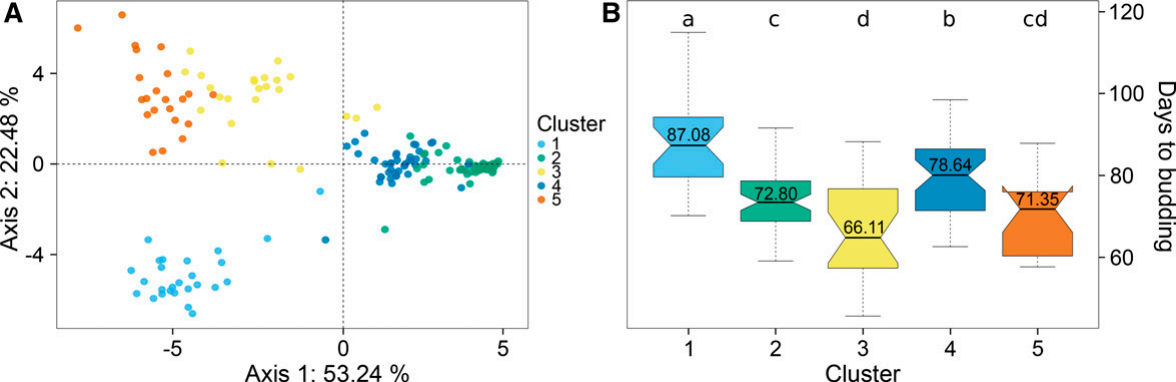
(A) Discriminant analysis of principal components plot for the five inferred clusters using the k-means algorithm (Jombart and Ahmed 2011). (B) Boxplots for number of days to budding for each DAPC-inferred cluster. Letters above boxplots display *Tukey*-test results. Clusters with the same letter are not significantly differentiated from each other. Values within boxplots display the mean time to budding for each cluster.

We inferred five genetic clusters (Figure 1A) by k-means clustering (Figure S1 in File S1). The clusters are differentiated by geographic origin and flowering time. Cluster 1 (*n* = 25) consists of geographically diverse accessions (Table S4 in File S1) without a distinct geographic origin, but differs from the other clusters by its high average time to flowering (Figure 1B). Clusters 2 (*n* = 56) and 4 (*n* = 40) consist of predominately European accessions that differ by their mean time to budding. Clusters 3 (*n* = 24) and 5 (*n* = 22) consist mainly of Asian accessions that also differ by mean time to budding.

**Figure 2:**
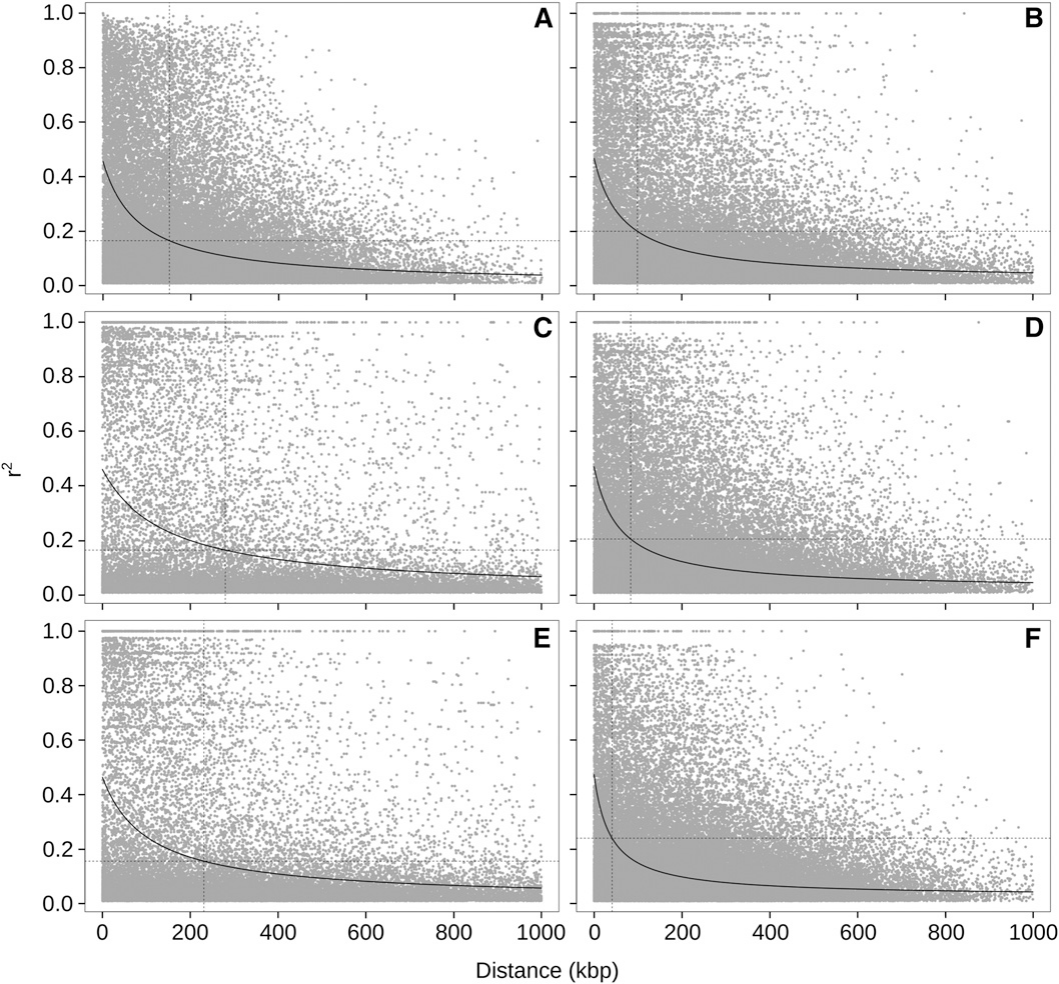
LD decay in the whole population (A) and clusters 1–5 (B–F). The dashed horizontal line indicates the average background LD of all chromosomes of a respective population. The dashed vertical line indicates the maximum distance between linked markers and is used as reference point for the LD decay.

We next tested whether the five clusters also differ by the extent of genome-wide LD, which may reflect differences in the breeding history. Based on the method described by Breseghello and Sorrells (2006), we estimated background LD as $r^{2}=0.17$ intersecting with the nonlinear regression curve at ∼151 kbp (Figure 2). The extent of LD is influenced by population structure and history, and we therefore calculated LD parameters for each cluster. Clusters 1, 3, and 5 show a rapid decay in comparison with the whole population, with an average background LD of $r^{2}=0.20,0.20$, and 0.24, extending to ∼98, ∼83, and ∼41 kbp (Figure 2). Clusters 2 and 4 have lower background LD values of 0.17 and 0.16, but higher long-range LD with averages of ∼280 and ∼231 kbp.

The average persistence of linkage phase is moderate between all clusters except for cluster 5, which reaches the expectation value of independent segregation (50%) for unlinked markers between clusters already at close distances in comparison with cluster 2 and cluster 4 (Figure S2 in File S1), indicating a stronger differentiation between those clusters.

### GWAS of six yield-related traits

Since the accessions show a strong population structure, we conducted a GWAS with models that correct for population structure and used the λ parameter to assess how well a correction was achieved. Overall, both GWAS methods showed λ values close to 1 (reflecting a good correction) for all traits and data (imputed *vs.* unimputed; Table S5 in File S1), which shows that the two methods account sufficiently well for population structure.

In total, 24 SNPs are associated with at least one of the six curd-related traits (Figure S3 in File S1; FDR=0.2). With EMMAX, six of 675 unimputed SNPs were associated with the traits of curd width, cluster width, number of branches, and number of days to budding. Only three SNPs of the imputed data set were associated with number of days to budding using EMMAX. Significant SNPs between the imputed and unimputed data sets differ from each other; using the imputed SNPs we identified an additional putative major QTL on chromosome O6 (Figure S4 in File S1 and Table 1). MLMM identified nine SNPs associated with apical length, number of branches, nearest branch length, or number of days to budding using the unimputed data set, whereas in total six SNPs from the imputed data set were associated with either apical length or number of days to budding (Table 1).

**Table 1:** Overview of significant associations detected with EMMAX and MLMM

				Rank		*B. oleracea*	*A. thaliana*		Variance explained		
Trait	Chr	Pos	Method	RR	BayesB	Gene ID	Ortholog	Description	Phenotypic	Genetic	MAF
Apical Length	2	26,787,029	MLMMI	30,559	29,378	Bol035969	AtSec6	Cell growth	4.1	37.3	0.05
Apical Length	3	20,986,662	MLMMI	2397	1712	Bol035507	AT1G53730	Protein coding	11.6	105.3	0.03
Apical Length	6	4,914,166	MLMMI	4902	1785	Bol032997	—	Protein kinase	10.6	96.0	0.08
Apical Length	6	34,494,415	MLMMU	2111	1194	Bol040102	AT1G71780	Biological processes	2.2	20.3	0.13
Cluster Width	2	5,063,181	EMMAXU	8	4	Bol007138	AT1G03220	Proteolysis	19.1	34.1	0.22
Curd Width	2	3,528,844	EMMAXU	1	1	Bol021232	AT5G19110	Heat stress	14.4	32.6	0.44
Curd Width	2	5,063,181	EMMAXU	6	4	Bol007138	AT1G03220	Proteolysis	12.1	27.4	0.22
Length of Nearest Branch	9	25,012,587	MLMMU	2	2	Bol012235	—	unknown	7.9	158.7	0.06
Number of Branches	6	2,323,306	EMMAXU	2	2	Bol035509	AT1G75310	Protein binding	6.2	23.7	0.06
Number of Branches	6	2,323,306	MLMMU	2	2	Bol035509	AT1G75310	Protein binding	6.2	23.7	0.06
Number of Branches	7	41.524,584	EMMAXU	1	1	Bol024369	AT2G17050	NBS gene family	8.5	32.6	0.21
Number of Branches	7	41,524,584	MLMMU	1	1	Bol024369	AT2G17050	NBS gene family	8.5	32.6	0.21
Number of Days to Budding	1	37,688,065	EMMAXU	1	1	Bol023068	AT3G09240	Signaling pathway	13.5	14.4	0.2
Number of Days to Budding	1	37,688,065	MLMMU	1	1	Bol023068	AT3G09240	Signaling pathway	13.5	14.4	0.2
Number of Days to Budding	2	2,708,156	MLMMU	5	6	Bol024638	AT5G10090	Flowering related	16.4	17.4	0.57
Number of Days to Budding	2	2,708,163	MLMMU	6	5	Bol024638	AT5G65160	Flowering related	16.4	17.4	0.57
Number of Days to Budding	2	2,708,182	MLMMU	7	4	Bol024638	AT5G65180	Flowering related	16.4	17.4	0.57
Number of Days to Budding	6	2,949,314	EMMAXI	1861	572	Bol026132	AT1G75010	Flowering	25.2	26.8	0.05
Number of Days to Budding	6	2,949,314	MLMMI	1861	572	Bol026132	AT1G75010	Flowering	25.2	26.8	0.05
Number of Days to Budding	7	936,738	EMMAXI	2	5	Bol027177	—	unknown	4.2	4.5	0.36
Number of Days to Budding	7	936,738	MLMMI	2	5	Bol027177	—	unknown	4.2	4.5	0.36
Number of Days to Budding	7	936,770	EMMAXI	1	1	Bol027177	—	unknown	6.4	6.8	0.23
Number of Days to Budding	7	936,770	MLMMI	1	1	Bol027177	—	unknown	6.4	6.8	0.23
Number of Days to Budding	7	41,524,584	MLMMU	2	3	Bol024369	AT2G17050	NBS gene family	4.1	4.4	0.21

The Rank column indicates which rank the significant association had among marker effects in the genomic prediction with ridge regression (RR) or BayesB methods. The last letter in the Method column indicates in which data set the QTL was discovered (U, unimputed, I, imputed). Phenotypic and genotypic variance indicate the percentage of variance explained by the respective SNP. Chr, chromosome; MAF, minor allele frequency; Pos, position.

Of the 24 SNPs significantly associated with the six traits, six QTL were identified by EMMAX and by MLMM. Moreover, one SNP (C02:5063181) was associated with both curd width and cluster width. Another SNP (C07:41524584) was associated with number of days to budding and number of branches (Table 1). We identified minor-effect QTL related to apical length, number of branches, and nearest branch length. Taken together, the results indicate that the imputation slightly increased the number of significantly associated SNPs.

Further, we tested whether SNPs identified as significantly associated with phenotypic variation also contribute to population structure (expressed as high DAPC loadings using only the imputed data; Figure S5 and S6 in File S1). For the traits of curd width and cluster width, no significant SNP detected by the GWAS was linked to a SNP with a high DAPC loading (Figure S5 in File S1), but for number of days to budding the significant association detected on chromosome O6 is located close to SNPs with high DAPC loadings (Figure S6 in File S1). This suggests that SNPs linked with flowering time variation also contribute to population differentiation.

### Genomic prediction with GBLUP and BayesB

Finally, we tested whether genomic prediction of phenotypic traits can be carried out with genetically diverse gene bank accessions to select new genetic resources for breeding purposes. The average prediction ability for each trait was calculated using five cross-validations with 10 replications; it ranged from 0.13 to 0.65 with GBLUP (Table 2) and from 0.09 to 0.66 with BayesB (Table 3) for the different traits and data sets. The prediction ability for RRBLUP was the same as for GBLUP, and we used RRBLUP estimates of marker effects for comparison with GWAS results. For all traits and both methods (GBLUP and BayesB), average prediction abilities were higher with imputed than with unimputed data, but the differences were minor (0.42 *vs.* 0.39).

**Table 2:** Prediction ability for six curd-related traits with different data sets using GBLUP

		Imputed Data			
Trait	Unimputed Data	BEAGLE	fastPHASE	Corrected	Mean
Curd Width	0.38	0.45	0.45	0.45	0.43
Cluster Width	0.62	0.65	0.65	0.59	0.63
Number of Branches	0.34	0.38	0.38	0.31	0.35
Apical Length	0.13	0.13	0.14	0.08	0.12
Nearest Branch	0.22	0.27	0.28	0.21	0.25
Number of Days	0.63	0.63	0.64	0.39	0.57
Mean	0.39	0.42	0.42	0.34	0.39

Unimputed: prediction ability using 675 SNPs. Imputed: prediction ability using BEAGLE and fastPHASE imputed data. Corrected: prediction ability for the GBLUP model with a realized relationship matrix corrected for population structure.

**Table 3:** Prediction ability for six curd-related traits with different data sets using BayesB

		Imputed Data		
Trait	Unimputed Data	BEAGLE	fastPHASE	Mean
Curd Width	0.35	0.40	0.44	0.40
Cluster Width	0.60	0.64	0.66	0.64
Number of Branches	0.38	0.35	0.41	0.38
Apical Length	0.09	0.12	0.10	0.10
Nearest Branch	0.23	0.28	0.29	0.26
Number of Days	0.66	0.66	0.61	0.64
Mean	0.39	0.41	0.42	0.40

Unimputed: prediction ability using 675 SNPs. Imputed: prediction ability using BEAGLE and fastPHASE imputed data.

With BayesB, prediction ability ranged from 0.09 to 0.66 (Table 3). Imputation resulted in slightly higher prediction abilities for all traits, except for number of days to budding. The prediction ability of fastPHASE was slightly higher than with BEAGLE for all traits except number of days to budding and apical length (Table 3). To compare the GWAS with the genomic prediction results, we ranked the marker effects estimated by RRBLUP and BayesB in descending order and compared them with the 24 significant associations detected by EMMAX and MLMM. Of these 24 SNPs, 18 also produced the largest marker effects and were among the top eight SNPs with the highest *P*-values in the GWAS (Table 1).

**Figure 3:**
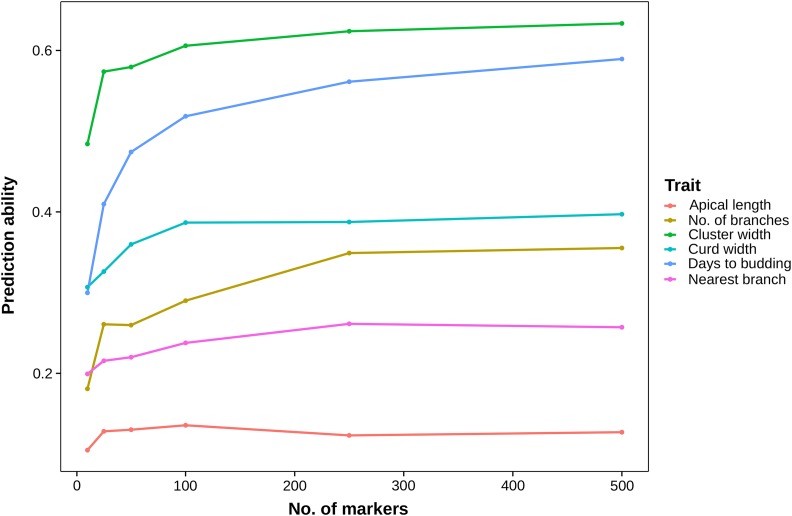
Effect of increasing the number of markers, included in a five-fold cross-validation with 10 replications using a standard GBLUP model, on prediction ability. Values represent averages of 100 runs. 10, 25, 50, 100, 250 and 500 markers, respectively, were sampled randomly for each run.

We assessed the influence of population structure on prediction ability using cross-validation. A correction for population structure resulted in a minor decrease in prediction ability for most traits, except curd width. The decrease in prediction ability was substantial for number of days to budding (0.64 to 0.39; Table 2). To test the influence of population structure on prediction ability, we randomly sampled subsets of markers and observed fairly high prediction abilities for small marker numbers (<100; Figure 3). Another approach to characterize the effect of population structure on prediction ability is to estimate the phenotypic variance explained by the first three principal components of a principal component analysis (Table S6 in File S1). According to this analysis, population structure had a strong effect on the genomic prediction ability of cluster width (40.01% variation explained by principal component 1), curd width (16.37% by principal component 1), and number of days to budding (27.66% by principal component 2). The variance explained by principal components was marginal for the other traits.

## Discussion

### Phenotypic variation

Our sample of cauliflower accessions could be grouped into distinct genetic clusters that also differed in phenotypic traits. Heritability estimates of the traits of cluster width, curd width, and number of days to budding were similar to previous studies (Lan and Paterson 2000; Matschegewski *et al.* 2015) and indicate a sufficient quality of the field trial data for GWAS and genomic prediction. Heritabilities for number of branches, apical length, and nearest branch were very low, which reflects a low data quality or a more complex genetic architecture.

### Strong population structure and differences in LD decay

A comparison of the population structure inferred by genetic markers and the passport data indicates limitations of available passport data. Among the 93 USDA accessions in the sample, 49 have a European origin according to their passport data. Of these, 24 (49%) cluster with the European accessions from IPK and the remaining 25 (51%) are distributed between cluster 1 (late flowering, geographically diverse accessions) and cluster 5 (predominately Asian accessions). This is unexpected under the assumption of a strong correlation between geographic origin and genetic relationship, but may be explained by incomplete passport data. Data about the seed donor but not the collection site were available for 79 of 93 USDA accessions (85%), and in these cases the true geographic origin could not be verified. For this reason, the analysis of genetic relationship allows a putative geographic origin of accessions to be assigned and can be used to complement missing passport data.

Genome-wide LD levels in the complete sample, which we measured as LD decay, were fairly high for an outcrossing species. The maximum physical distance for genetic linkage is reached at 151 kbp using the whole population. This value is comparable with that of self-fertilizing species, but lower than a previous estimate for cauliflower of up to 400 kbp (Matschegewski *et al.* 2015). A comparison of LD decay and background LD for the complete sample and each inferred cluster revealed differences between these groups. For example, the high LD in clusters 2 and 4 that consist mainly of European cultivars suggests that breeding and small population size likely contributed to the observed differences.

The highest level of long-range LD is located on chromosome O4 in accessions of clusters 2 and 4 (data not shown). This chromosome harbors multiple paralogs of the *BolbZIP* gene family, which is involved into cold stress tolerance (Hwang *et al.* 2016). Although this trait was not evaluated in our field trials, we hypothesize that selection for cold tolerance in European varieties may explain the higher level of LD.

Finally, we compared the persistence of linkage phase between clusters (Figure S2 in File S1). The proportion of markers in the same linkage phase was moderate between most clusters, but slightly lower between clusters 2 and 5 and clusters 4 and 5, which indicates that genomic prediction ability is mainly influenced by the genetic relationship of individuals and not by a persistent linkage of marker alleles with causal QTL in different clusters.

### Significant marker–trait associations in a GWAS of traits with a high heritability

The phenotypic differences in flowering time and curd width between genetic clusters suggest a genetic basis for these differences. We used two GWAS methods for the imputed and unimputed SNP data to map QTL controlling these traits and identified 24 SNPs that were associated with at least one of the six phenotypic traits. The genomic locations of SNPs associated with flowering time (*e.g.*, time to budding) coincide well with QTL regions identified in previous studies. For example, a SNP on chromosome O6 explained 26.8% of variation and was close to a QTL detected by Hasan *et al.* (2016). Another SNP found on chromosome O2 explains 16.4% of the phenotypic variation for the same trait and maps to a known QTL (Okazaki *et al.* 2007; Uptmoor *et al.* 2008; Matschegewski *et al.* 2015). The last of these studies also demonstrated the influence of QTL on chromosome O2 on time to flowering. The QTL region harbors a homolog of the *A. thaliana Flowering Locus C* (*FLC*) gene, which controls vernalization and has a major influence on flowering time.

A third SNP on chromosome O1 explained 13.5% of genetic variation. It is located in a genomic region that harbors an ortholog to the *A. thaliana* the flowering time gene *VRN1* (Matschegewski *et al.* 2015). Additional SNPs that explain a minor proportion of the genetic variation for flowering time are located on chromosome O7 but have not been described in literature.

The GWAS also identified three polymorphisms on chromosome O2 that are associated with curd width and cluster width. Strongly associated polymorphisms overlapped for these traits, which can be explained by the fact that the two traits are highly correlated with each other (Table S3 in File S1). The SNPs explain between 27.4 and 34.1% of the genetic variation and therefore seem to be linked to a major QTL. These SNPs are located in genes whose *A. thaliana* orthologs regulate response to heat stress, which is consistent with the observation that temperature has a strong effect on cauliflower curd development (Matschegewski *et al.* 2015). Hasan *et al.* (2016) identified a QTL on the same chromosome (O2), which was only expressed under high-temperature conditions (27°).

The differences between the two GWAS methods (EMMAX and MLMM) may result from the small sample size. Sample size has a great influence on detection power for complex traits in GWAS (Korte and Farlow 2013). However, small samples are sufficient to detect major QTL, because simulations show that QTL explaining 10% of genetic variance can be identified in a sample of 100 genotypes (Gawenda *et al.* 2015). As an example, a flowering time QTL caused by variation in the vernalization response gene *FRIGIDA* was found in a sample of only 107 natural accessions of *A. thaliana* (Atwell *et al.* 2010). We therefore consider our sample of 174 individuals sufficiently large to reliably detect major QTL for highly heritable traits such as flowering time regulation, curd width, and cluster width.

### Evaluation of genomic prediction in cauliflower genetic resources

Genomic prediction is a useful method for characterizing gene bank accessions, because it may allow phenotyping to be restricted to a subset of accessions in order to predict trait values for the complete collection. We used GBLUP and BayesB for prediction, because these models have a good performance, stable prediction ability, and are suited for different genetic architectures (Meuwissen *et al.* 2001; Heslot *et al.* 2012). Genomic prediction worked best for traits with a high heritability such as number of days to budding and cluster width, and less well for the traits of apical length and length of nearest branch (Table 2 and Table 3). In addition to a low heritability, the precise phenotyping of the latter two traits is challenging, and measurement errors result in biased estimates of variance components and adjusted means that affect prediction ability.

Since this is the first study that uses genomic prediction in cauliflower, we can only compare our results with those for other *Brassica* species. Prediction abilities for number of days to budding and curd width are similar to those for flowering time (0.70) and grain yield (0.50) in rapeseed (*B. napus*) (Würschum *et al.* 2014), a close relative of *B. oleracea*, which suggests that genomic selection is a robust and promising breeding method for *Brassica* species. Prediction ability is influenced by the presence of population structure (Thorwarth *et al.* 2017). If both training and validation sets for genomic selection show a population structure, a correction for structure reduces prediction ability (Guo *et al.* 2014). The effect of population structure on prediction ability depends on the trait. For the trait days to budding, prediction ability decreased from 0.64 to 0.39 after correction, whereas a correction had no effect on prediction ability of the other five traits.

The strong effect of structure correction on prediction ability for flowering is consistent with our observation that this trait differs significantly between genetic clusters. Mainly loci close to the putative QTL on chromosome O6 seem to contribute to population differentiation (measured as high loading values in the DAPC analysis) and are close to SNPs linked to flowering time QTL in the GWAS. This indicates that selection for different flowering time or adaption to different areas contributed to the genetic population structure (Matschegewski *et al.* 2015). For the other traits such as curd with or cluster width there is no such overlap, which may be explained by a more complex genetic architecture of these traits.

Genomic prediction models simultaneously utilize all SNPs for calculating the breeding value. RRBLUB and BayesB estimate marker effects, which can be used for comparison with GWAS models that consider each SNP separately. We found strong overlaps of significant SNPs in the GWAS, with the highest marker effects obtained from the genomic prediction models (Table 1). This overlap reflects the similarity of the statistical models used for GWAS and genomic prediction, and suggests that these SNPs are linked to robust QTL. In summary, a comparison of putative causal SNPs identified by GWAS, the estimation of marker effects in genomic prediction, and the analysis of allele contribution to population structure (DAPC loading) may be considered as validation of significant marker–trait associations and provides useful information to improve genetic analysis (Spindel *et al.* 2016; Bian and Holland 2017).

The success of genomic prediction depends on the method used and the size of the training set. We observed only minor differences between GBLUP and BayesB regarding their mean prediction ability over all traits and data sets (Table 2 and Table 3). This observation confirms earlier work that Bayesian models do not outperform GBLUP (Bao *et al.* 2014). Since GBLUP is computationally less expensive and much simpler to implement than BayesB, our results suggest that GBLUP can be recommended for genomic selection in cauliflower breeding. The size of the training set is another point to consider, because larger training sets improve prediction ability and allow a robust estimation of marker effects (Windhausen *et al.* 2012). We provide a first assessment of genomic prediction in a small sample of genetically diverse cauliflower gene bank material, but larger training sets are required, especially for traits with a complex genetic architecture (>1000; Thorwarth *et al.* 2017).

### Imputation effect on GWAS and genomic prediction results

Although GBS is an efficient method for obtaining large numbers of polymorphisms, the resulting data have a high proportion of missing values. Imputation of missing data may be used to overcome this limitation. In our GWAS analysis, we obtained fewer significant associations (10) with the imputed data than with unimputed data (14; Figure S3 in File S1). However, imputation may improve the power of GWAS because in the imputed data only we identified one additional QTL for flowering time on chromosome O6, which was also found by Hasan *et al.* (2016). However, several QTL observed in the unimputed data were not identified in the imputed data. One explanation for this discrepancy is significance levels that are based on marker number. For quantitative traits that are influenced by QTL with small effects, a Bonferroni correction of *P*-values in GWAS may be too conservative and result in a high proportion of false negatives (Perneger 1998). Therefore, we used the FDR, which is the expected proportion of significant associations that are false positives. It is defined as $\frac{i}{n}\times Q$, where *i* is the rank of the ascending *P*-values, *n* is the number of markers (tests), and *Q* is the FDR (Benjamini and Hochberg 1995). We used a FDR of 0.05, which implies that 20% of the observed significant associations are false positives. A smaller or higher *Q* value will lead to a more stringent or a more relaxed threshold, respectively. For example, with an FDR value 5% we observed 10 and with an FDR of 30% we obtained a total of 35 significant associations with EMMAX and MLMM. It should be noted that FDR thresholds are specific for each combination of traits and data sets, and for this reason the identification and implementation of optimal significance thresholds is still debated (Sham and Purcell 2014). Thus, a careful assessment of different thresholds together with the ranking of marker effects and a comparison of the DAPC loadings can help to improve the conclusions drawn from GWAS studies and validate the robustness of putative QTL.

The difference in the number of significant associations between the unimputed and imputed data sets is also influenced by the imputation method. The fastPHASE method is not well suited for GBS data, because a parameter vector of allele frequencies has to be estimated for each SNP. As fastPHASE performs best with SNPs genotyped in many individuals (Scheet and Stephens 2006), the high proportion of missing data reduces the average number of individuals available per SNP. As the second imputation method, we used BEAGLE, which performs well with medium to large sample sizes (>1000 individuals), but not as well as fastPHASE when compared for small samples of 100 individuals, as demonstrated by Browning and Browning (2011). The clustering approach of BEAGLE flexibly changes cluster numbers to better accommodate local LD patterns, but in general neither method can cope very well with a large amount of missing data as observed in this study (Yang *et al.* 2014; Xavier *et al.* 2016).

The imputation of missing markers slightly improved prediction ability for most traits, consistent with previous studies that revealed only minor advantages of imputation, particularly in self-fertilizing crops with a slow LD decay (Rutkoski *et al.* 2013; Poland *et al.* 2012a; Jarquín *et al.* 2014). Prediction ability is mainly determined by relatedness and less by linkage between marker and QTL, as indicated by the linkage phase analysis. For this reason, imputation has little influence on prediction ability, because small numbers of high-quality SNPs are sufficient to capture the relationship structure among individuals.

In summary, there was only a minor advantage of imputation for GWAS and genomic prediction with our sample. However, the rapid development of genome sequencing technologies and rapidly decreasing costs will alleviate the problem of missing data for genomic prediction, as genome resequencing will uncover most genetic variation, in particular for crops with small genome sizes such as *B. oleracea* (Golicz *et al.* 2016).

### Implications for the utilization of genomic resources

Breeding populations of cauliflower are characterized by a low genetic diversity (Golicz *et al.* 2016), which limits the potential for improving varieties that meet the expectations of growers and consumers. We showed that both GWAS and genomic prediction contribute to the genetic analysis of complex traits and to the identification of novel and potentially useful genetic variation in gene bank material (Yu *et al.* 2016). Our study also provides a perspective with respect to the utilization of *ex situ* conserved gene bank accessions. Our sample was randomly selected and represents a broad genetic and geographic diversity. We achieved reasonable genomic prediction abilities, although genetic clustering inflates prediction ability if the cluster structure is correlated with trait distribution. For this reason, genotyping whole collections of gene bank accessions and the phenotyping of a sufficiently large subset allows the prediction of relevant phenotypic traits in the whole collection and the subsequent selection of accessions for further use as genetic resources. This will contribute to an efficient description and utilization of *ex situ* conserved germplasm resources.

## Supplementary Material

Supplemental material is available online at www.g3journal.org/lookup/suppl/doi:10.1534/g3.117.300199/-/DC1.

## References

[bib1] Atwell S. Huang Y. S. Vilhjálmsson B. J. Willems G. Horton M., 2010 Genome-wide association study of 107 phenotypes in a common set of Arabidopsis thaliana inbred lines. Nature 465: 627–631. 20336072 10.1038/nature08800 PMC3023908

[bib2] Bao Y. Vuong T. Meinhardt C. Tiffin P. Denny R., 2014 Potential of association mapping and genomic selection to explore PI 88788 derived soybean cyst nematode resistance. Plant Genome 7 DOI:10.3835/plantgenome2013.11.0039

[bib3] Bates D. Maechler M. Bolker B. Walker S., 2014 lme4: linear mixed-effects models using Eigen and S4. R package version 1: 1–23.

[bib4] Benjamini Y. Hochberg Y., 1995 Controlling the false discovery rate: a practical and powerful approach to multiple testing. J. R. Stat. Soc. B 57: 289–300.

[bib5] Bevan M. W. Uauy C. Wulff B. B. Zhou J. Krasileva K., 2017 Genomic innovation for crop improvement. Nature 543: 346–354. 28300107 10.1038/nature22011

[bib6] Bian Y. Holland J., 2017 Enhancing genomic prediction with genome-wide association studies in multiparental maize populations. Heredity 118: 585–593. 28198815 10.1038/hdy.2017.4 PMC5436027

[bib7] Breseghello F. Sorrells M. E., 2006 Association mapping of kernel size and milling quality in wheat (Triticum aestivum L.) cultivars. Genetics 172: 1165–1177. 16079235 10.1534/genetics.105.044586 PMC1456215

[bib8] Browning S. R. Browning B. L., 2007 Rapid and accurate haplotype phasing and missing-data inference for whole-genome association studies by use of localized haplotype clustering. Am. J. Hum. Genet. 81: 1084–1097. 17924348 10.1086/521987 PMC2265661

[bib9] Browning S. R. Browning B. L., 2011 Haplotype phasing: existing methods and new developments. Nat. Rev. Genet. 12: 703–714. 21921926 10.1038/nrg3054 PMC3217888

[bib10] Cai D. Xiao Y. Yang W. Ye W. Wang B., 2014 Association mapping of six yield-related traits in rapeseed (Brassica napus L.). Theor. Appl. Genet. 127: 85–96. 24121524 10.1007/s00122-013-2203-9

[bib11] Conomos M. P. Reiner A. P. Weir B. S. Thornton T. A., 2016 Model-free estimation of recent genetic relatedness. Am. J. Hum. Genet. 98: 127–148. 26748516 10.1016/j.ajhg.2015.11.022 PMC4716688

[bib12] Crossa J. Campos G. l. Perez P. Gianola D. Burgueno J., 2010 Prediction of genetic values of quantitative traits in plant breeding using pedigree and molecular markers. Genetics 186: 713–724. 20813882 10.1534/genetics.110.118521 PMC2954475

[bib13] Crossa J. Beyene Y. Kassa S. Perez P. Hickey J. M., 2013 Genomic prediction in maize breeding populations with genotyping-by-sequencing. G3(Bethesda) 3: 1903–1926. 24022750 10.1534/g3.113.008227 PMC3815055

[bib14] De Roos A. P. W. Hayes B. J. Spelman R. J. Goddard M. E., 2008 Linkage disequilibrium and persistence of phase in Holstein-Friesian, Jersey and Angus cattle. Genetics 179: 1503–1512. 18622038 10.1534/genetics.107.084301 PMC2475750

[bib15] Devlin B. Roeder K., 1999 Genomic control for association studies. Biometrics 55: 997–1004. 11315092 10.1111/j.0006-341x.1999.00997.x

[bib16] Edae E. A. Byrne P. F. Haley S. D. Lopes M. S. Reynolds M. P., 2014 Genome-wide association mapping of yield and yield components of spring wheat under contrasting moisture regimes. Theor. Appl. Genet. 127: 791–807. 24408378 10.1007/s00122-013-2257-8

[bib17] Elshire R. J. Glaubitz J. C. Sun Q. Poland J. A. Kawamoto K., 2011 A robust, simple genotyping-by-sequencing (GBS) approach for high diversity species. PLoS One 6: e19379. 21573248 10.1371/journal.pone.0019379 PMC3087801

[bib18] Fu Y.-B. Cheng B. Peterson G. W., 2014 Genetic diversity analysis of yellow mustard (Sinapis alba L.) germplasm based on genotyping by sequencing. Genet. Resour. Crop Evol. 61: 579–594.

[bib19] Gawenda I. Thorwarth P. Günther T. Ordon F. Schmid K. J., 2015 Genome-wide association studies in elite varieties of German winter barley using single-marker and haplotype-based methods. Plant Breed. 134: 28–39.

[bib20] Golicz A. A. Bayer P. E. Barker G. C. Edger P. P. Kim H., 2016 The pangenome of an agronomically important crop plant Brassica oleracea. Nat. Commun. 7: 13390. 27834372 10.1038/ncomms13390 PMC5114598

[bib21] Guo Z. Tucker D. M. Basten C. J. Gandhi H. Ersoz E., 2014 The impact of population structure on genomic prediction in stratified populations. Theor. Appl. Genet. 127: 749–762. 24452438 10.1007/s00122-013-2255-x

[bib22] Habier D. Fernando R. L. Garrick D. J., 2013 Genomic BLUP decoded: a look into the black box of genomic prediction. Genetics 194: 597–607. 23640517 10.1534/genetics.113.152207 PMC3697966

[bib23] Hasan M. Friedt W. Pons-Kühnemann J. Freitag N. M. Link K., 2008 Association of gene-linked SSR markers to seed glucosinolate content in oilseed rape (Brassica napus ssp. napus). Theor. Appl. Genet. 116: 1035–1049. 18322671 10.1007/s00122-008-0733-3

[bib24] Hasan Y. Briggs W. Matschegewski C. Ordon F. Stützel H., 2016 Quantitative trait loci controlling leaf appearance and curd initiation of cauliflower in relation to temperature. Theor. Appl. Genet. 129: 1273–1288. 26993486 10.1007/s00122-016-2702-6

[bib25] Hawkes J., 1991 The importance of genetic resources in plant breeding. Biological Journal of the Linncan Society 43: 3–10.

[bib26] Hayes B. Bowman P. Chamberlain A. Goddard M., 2009 Invited review: genomic selection in dairy cattle: progress and challenges. J. Dairy Sci. 92: 433–443. 19164653 10.3168/jds.2008-1646

[bib27] Heffner E. L. Sorrells M. E. Jannink J.-L., 2009 Genomic selection for crop improvement. Crop Sci. 49: 1.

[bib28] Heffner E. L. Lorenz A. J. Jannink J.-L. Sorrells M. E., 2010 Plant breeding with genomic selection: gain per unit time and cost. Crop Sci. 50: 1681.

[bib29] Heslot N. Yang H.-P. Sorrells M. E. Jannink J.-L., 2012 Genomic selection in plant breeding: a comparison of models. Crop Sci. 52: 146.

[bib30] Hwang I. Manoharan R. K. Kang J.-G. Chung M.-Y. Kim Y.-W., 2016 Genome-wide identification and characterization of bzip transcription factors in Brassica oleracea under cold stress. BioMed Res. Int. 2016: 4376598. 27314020 10.1155/2016/4376598 PMC4893578

[bib31] Jannink J.-L. Lorenz A. J. Iwata H., 2010 Genomic selection in plant breeding: from theory to practice. Brief. Funct. Genomics 9: 166–177. 20156985 10.1093/bfgp/elq001

[bib32] Jarquín D. Kocak K. Posadas L. Hyma K. Jedlicka J., 2014 Genotyping by sequencing for genomic prediction in a soybean breeding population. BMC Genomics 15: 740. 25174348 10.1186/1471-2164-15-740 PMC4176594

[bib33] Jestin C. Lodé M. Vallée P. Domin C. Falentin C., 2011 Association mapping of quantitative resistance for Leptosphaeria maculans in oilseed rape (Brassica napus L.). Mol. Breed. 27: 271–287.

[bib34] Jombart T. Ahmed I., 2011 Adegenet 1.3–1: new tools for the analysis of genome-wide SNP data. Bioinformatics 27: 3070–3071. 21926124 10.1093/bioinformatics/btr521 PMC3198581

[bib35] Kang H. M. Sul J. H. Service S. K. Zaitlen N. A. Kong S.-y., 2010 Variance component model to account for sample structure in genome-wide association studies. Nat. Genet. 42: 348–354. 20208533 10.1038/ng.548 PMC3092069

[bib36] Korte A. Farlow A., 2013 The advantages and limitations of trait analysis with gwas: a review. Plant Methods 9: 29. 23876160 10.1186/1746-4811-9-29 PMC3750305

[bib37] König S. Simianer H. Willam A., 2009 Economic evaluation of genomic breeding programs. J. Dairy Sci. 92: 382–391. 19109296 10.3168/jds.2008-1310

[bib38] Lan T.-H. Paterson A. H., 2000 Comparative mapping of quantitative trait loci sculpting the curd of Brassica oleracea. Genetics 155: 1927–1954. 10924486 10.1093/genetics/155.4.1927 PMC1461177

[bib39] Li F. Chen B. Xu K. Wu J. Song W., 2014 Genome-wide association study dissects the genetic architecture of seed weight and seed quality in rapeseed (Brassica napus L.). DNA Res. 21: 355–367. 24510440 10.1093/dnares/dsu002 PMC4131830

[bib40] Li H. Durbin R., 2009 Fast and accurate short read alignment with Burrows-Wheeler transform. Bioinformatics 25: 1754–1760. 19451168 10.1093/bioinformatics/btp324 PMC2705234

[bib41] Li H. Peng Z. Yang X. Wang W. Fu J., 2012 Genome-wide association study dissects the genetic architecture of oil biosynthesis in maize kernels. Nat. Genet. 45: 43–50. 23242369 10.1038/ng.2484

[bib42] Li H. Liu Q. Zhang Q. Qin E. Jin C., 2017 Curd development associated gene (cdag1) in cauliflower (Brassica oleracea l. var. botrytis) could result in enlarged organ size and increased biomass. Plant Sci. 254: 82–94. 27964787 10.1016/j.plantsci.2016.10.009

[bib43] Liu S. Liu Y. Yang X. Tong C. Edwards D., 2014 The Brassica oleracea genome reveals the asymmetrical evolution of polyploid genomes. Nat. Commun. 5: 3930. 24852848 10.1038/ncomms4930 PMC4279128

[bib44] Longin C. F. H. Reif J. C., 2014 Redesigning the exploitation of wheat genetic resources. Trends Plant Sci. 19: 631–636. 25052155 10.1016/j.tplants.2014.06.012

[bib45] Marchini J. Howie B., 2010 Genotype imputation for genome-wide association studies. Nat. Rev. Genet. 11: 499–511. 20517342 10.1038/nrg2796

[bib46] Matschegewski C. Zetzsche H. Hasan Y. Leibeguth L. Briggs W., 2015 Genetic variation of temperature-regulated curd induction in cauliflower: elucidation of floral transition by genome-wide association mapping and gene expression analysis. Front. Plant Sci. 6: 720. 26442034 10.3389/fpls.2015.00720 PMC4564693

[bib47] Meuwissen T. H. Hayes B. J. Goddard M. E., 2001 Prediction of total genetic value using genome-wide dense marker maps. Genetics 157: 1819–1829. 11290733 10.1093/genetics/157.4.1819 PMC1461589

[bib48] Morris G. P. Rhodes D. H. Brenton Z. Ramu P. Thayil V. M., 2013 Dissecting genome-wide association signals for loss-of-function phenotypes in sorghum flavonoid pigmentation traits. G3(Bethesda) 3: 2085–2094. 24048646 10.1534/g3.113.008417 PMC3815067

[bib49] Norton G. J. Douglas A. Lahner B. Yakubova E. Guerinot M. L., 2014 Genome wide association mapping of grain arsenic, copper, molybdenum and zinc in rice (Oryza sativa L.) grown at four international field sites. PLoS One 9: e89685. 24586963 10.1371/journal.pone.0089685 PMC3934919

[bib50] Nyquist W. E. Baker R., 1991 Estimation of heritability and prediction of selection response in plant populations. Crit. Rev. Plant Sci. 10: 235–322.

[bib51] Okazaki K. Sakamoto K. Kikuchi R. Saito A. Togashi E., 2007 Mapping and characterization of FLC homologs and QTL analysis of flowering time in Brassica oleracea. Theor. Appl. Genet. 114: 595–608. 17136371 10.1007/s00122-006-0460-6

[bib52] Pei Y.-F. Li J. Zhang L. Papasian C. J. Deng H.-W., 2008 Analyses and comparison of accuracy of different genotype imputation methods. PLoS One 3: e3551. 18958166 10.1371/journal.pone.0003551 PMC2569208

[bib53] Pérez P. de los Campos G., 2014 Genome-wide regression and prediction with the BGLR statistical package. Genetics 198: 483–495. 25009151 10.1534/genetics.114.164442 PMC4196607

[bib54] Perneger T. V., 1998 What’s wrong with Bonferroni adjustments. BMJ 316: 1236–1238. 9553006 10.1136/bmj.316.7139.1236 PMC1112991

[bib55] Picchi V. Migliori C. Scalzo R. L. Campanelli G. Ferrari V., 2012 Phytochemical content in organic and conventionally grown Italian cauliflower. Food Chem. 130: 501–509. 10.1021/jf4026844 24134670

[bib56] Poland J. Endelman J. Dawson J. Rutkoski J. Wu S., 2012 a Genomic selection in wheat breeding using genotyping-by-sequencing. The Plant Genome Journal 5: 103.

[bib57] Poland J. A. Rife T. W., 2012 Genotyping-by-sequencing for plant breeding and genetics. The Plant Genome Journal 5: 92.

[bib58] Poland J. A. Brown P. J. Sorrells M. E. Jannink J.-L., 2012 b Development of high-density genetic maps for barley and wheat using a novel two-enzyme genotyping-by-sequencing approach. PLoS One 7: e32253. 22389690 10.1371/journal.pone.0032253 PMC3289635

[bib59] Purcell S. Neale B. Todd-Brown K. Thomas L. Ferreira M. A. R., 2007 PLINK: a tool set for whole-genome association and population-based linkage analyses. Am. J. Hum. Genet. 81: 559–575. 17701901 10.1086/519795 PMC1950838

[bib60] R Core Team, 2015 *R: A Language and Environment for Statistical Computing.* R Foundation for Statistical Computing, Vienna.

[bib61] Rezaeizad A. Wittkop B. Snowdon R. Hasan M. Mohammadi V., 2011 Identification of QTLs for phenolic compounds in oilseed rape (Brassica napus L.) by association mapping using SSR markers. Euphytica 177: 335–342.

[bib62] Romay M. C. Millard M. J. Glaubitz J. C. Peiffer J. A. Swarts K. L., 2013 Comprehensive genotyping of the USA national maize inbred seed bank. Genome Biol. 14: 1. 10.1186/gb-2013-14-6-r55 PMC3707059 23759205

[bib63] Rutkoski J. E. Poland J. Jannink J.-L. Sorrells M. E., 2013 Imputation of unordered markers and the impact on genomic selection accuracy. G3(Bethesda) 3: 427–439. 23449944 10.1534/g3.112.005363 PMC3583451

[bib64] Schaeffer L. R., 2006 Strategy for applying genome-wide selection in dairy cattle. J. Anim. Breed. Genet. 123: 218–223. 16882088 10.1111/j.1439-0388.2006.00595.x

[bib65] Scheet P. Stephens M., 2006 A fast and flexible statistical model for large-scale population genotype data: applications to inferring missing genotypes and haplotypic phase. Am. J. Hum. Genet. 78: 629–644. 16532393 10.1086/502802 PMC1424677

[bib66] Schmid K. Thorwarth P., 2014 Genomic selection in barley breeding. Springer Berlin Heidelberg 69: 367–378.

[bib67] Segura V. Vilhjálmsson B. J. Platt A. Korte A. Seren U., 2012 An efficient multi-locus mixed-model approach for genome-wide association studies in structured populations. Nat. Genet. 44: 825–830. 22706313 10.1038/ng.2314 PMC3386481

[bib68] Sham P. C. Purcell S. M., 2014 Statistical power and significance testing in large-scale genetic studies. Nat. Rev. Genet. 15: 335–346. 24739678 10.1038/nrg3706

[bib69] Sonah H. Bastien M. Iquira E. Tardivel A. Légaré G., 2013 An improved genotyping by sequencing (GBS) approach offering increased versatility and efficiency of SNP discovery and genotyping. PLoS One 8: e54603. 23372741 10.1371/journal.pone.0054603 PMC3553054

[bib70] Spindel J. Begum H. Akdemir D. Collard B. Redona E., 2016 Genome-wide prediction models that incorporate de novo gwas are a powerful new tool for tropical rice improvement. Heredity 116: 395. 26860200 10.1038/hdy.2015.113 PMC4806696

[bib71] Tardivel A. Sonah H. Belzile F. O’Donoughue L. S., 2014 Rapid identification of alleles at the soybean maturity gene E3 using genotyping by sequencing and a haplotype-based approach. Plant Genome 7 DOI:10.3835/plantgenome2013.10.0034

[bib72] Tester M. Langridge P., 2010 Breeding technologies to increase crop production in a changing world. Science 327: 818–822. 20150489 10.1126/science.1183700

[bib73] Thorwarth P. Ahlemeyer J. Bochard A.-M. Krumnacker K. Blümel H., 2017 Genomic prediction ability for yield-related traits in German winter barley elite material. Theor. Appl. Genet. 130: 1669–1683. 28534096 10.1007/s00122-017-2917-1

[bib74] Uptmoor R. Schrag T. Stützel H. Esch E., 2008 Crop model based QTL analysis across environments and QTL based estimation of time to floral induction and flowering in Brassica oleracea. Mol. Breed. 21: 205–216.

[bib75] Utz H. F. Melchinger A. E. Schön C. C., 2000 Bias and sampling error of the estimated proportion of genotypic variance explained by quantitative trait loci determined from experimental data in maize using cross validation and validation with independent samples. Genetics 154: 1839–1849. 10866652 PMC1461020

[bib76] VanLiere J. Rosenberg N., 2008 Mathematical properties of the *r*^2^ measure of linkage disequilibrium. Theor. Popul. Biol. 74: 130–137. 18572214 10.1016/j.tpb.2008.05.006 PMC2580747

[bib77] Wimmer V. Albrecht T. Auinger H.-J. Schon C.-C., 2012 Synbreed: a framework for the analysis of genomic prediction data using R. Bioinformatics 28: 2086–2087. 22689388 10.1093/bioinformatics/bts335

[bib78] Windhausen V. S. Atlin G. N. Hickey J. M. Crossa J. Jannink J.-L., 2012 Effectiveness of genomic prediction of maize hybrid performance in different breeding populations and environments. Genetics 2: 1427–1436. 10.1534/g3.112.003699 PMC3484673 23173094

[bib79] Würschum T. Abel S. Zhao Y., 2014 Potential of genomic selection in rapeseed (*Brassica napus* L.) breeding. Plant Breed. 133: 45–51.

[bib80] Xavier A. Muir W. M. Rainey K. M., 2016 Impact of imputation methods on the amount of genetic variation captured by a single-nucleotide polymorphism panel in soybeans. BMC Bioinformatics 17: 55. 26830693 10.1186/s12859-016-0899-7 PMC4736474

[bib81] Yang Y. Wang Q. Chen Q. Liao R. Zhang X., 2014 A new genotype imputation method with tolerance to high missing rate and rare variants. PLoS One 9: e101025. 24972110 10.1371/journal.pone.0101025 PMC4074155

[bib82] Yousef E. A. A. Lampei C. Schmid K. J., 2015 Evaluation of cauliflower genebank accessions under organic and conventional cultivation in Southern Germany. Euphytica 201: 389–400.

[bib83] Yousef T. Müller T. Schmid K. J., 2017 Comparative analysis of genetic diversity and differentiation of cauliflower (Brassica oleracea var. botrytis) accessions from two ex situ genebanks. bioRxiv. https//.org/10.1101/238840 10.1371/journal.pone.0192062 PMC5805252 29420661

[bib84] Yu X. Li X. Guo T. Zhu C. Wu Y., 2016 Genomic prediction contributing to a promising global strategy to turbocharge gene banks. Nat. Plants 2: 16150. 27694945 10.1038/nplants.2016.150

[bib85] Zhao Z. Gu H. Sheng X. Yu H. Wang J., 2016 Genome-wide single-nucleotide polymorphisms discovery and high-density genetic map construction in cauliflower using specific-locus amplified fragment sequencing. Front. Plant Sci. 7: 334. 27047515 10.3389/fpls.2016.00334 PMC4800193

[bib86] Zou J. Jiang C. Cao Z. Li R. Long Y., 2010 Association mapping of seed oil content in Brassica napus and comparison with quantitative trait loci identified from linkage mapping. Genome 53: 908–916. 21076506 10.1139/G10-075

