# Supplementary material for: Genomic Prediction and Association Mapping of Curd-Related Traits in Gene Bank Accessions of Cauliflower
Source: G3 (Bethesda). 2017 Dec 18;8(2):707–18. doi: 10.1534/g3.117.300199 (PMC5919744; doi:10.1534/g3.117.300199)
Supplement: Supplementary file 1 [file 707FileS1.pdf]

Genomic prediction and association mapping of curd-related traits in genebank accessions of cauliflower

Patrick Thorwarth Eltohamy A.A. Yousef Karl J. Schmid

December 14, 2017

Table S1 Genbank accession informations and DAPC cluster classification.

Accession	Type	Year of collection	Genebank	Origin	DAPC_cluster
PI 165488	Collector	1948	USDA	Asia	5
PI 169034	Collector	1948	USDA	Asia	5
PI 183214	Collector	1949	USDA	Africa	1
PI 204758	Unverified	1953	USDA	Europ	4
PI 204759	Unverified	1953	USDA	Europ	4
PI 204761	Unverified	1953	USDA	Europ	3
PI 204762	Unverified	1953	USDA	Europ	1
PI 204763	Unverified	1953	USDA	Europ	4
PI 204764	Unverified	1953	USDA	Europ	4
PI 204765	Unverified	1953	USDA	Europ	1
PI 204767	Unverified	1953	USDA	Europ	2
PI 204768	Unverified	1953	USDA	Europ	2
PI 204769	Unverified	1953	USDA	Europ	1
PI 204772	Unverified	1953	USDA	Europ	1
PI 204774	Unverified	1953	USDA	Europ	5
PI 204775	Unverified	1953	USDA	Europ	1
PI 204777	Unverified	1953	USDA	Europ	2
PI 204782	Unverified	1953	USDA	Europ	2
PI 208474	Unverified	1953	USDA	Europ	2
PI 208478	Unverified	1953	USDA	Europ	2
PI 208481	Unverified	1953	USDA	Europ	2
PI 208482	Unverified	1953	USDA	Europ	2
PI 208483	Unverified	1953	USDA	Europ	2
PI 208484	Unverified	1953	USDA	Europ	3
PI 209751	Unverified	1953	USDA	Europ	4
PI 209756	Unverified	1953	USDA	Europ	1
PI 209757	Unverified	1953	USDA	Europ	4
PI 209758	Unverified	1953	USDA	Europ	3
PI 212592	Cultivar	1953	USDA	Asia	3
PI 217934	Unverified	1954	USDA	Asia	4
PI 225852	Unverified	1955	USDA	Europ	2
PI 226504	Unverified	1955	USDA	Asia	5
PI 231048	Unverified	1956	USDA	Asia	1
PI 231207	Unverified	1956	USDA	Europ	1
PI 231209	Cultivar	1956	USDA	Europ	5
PI 232070	Unverified	1956	USDA	Africa	3
PI 234599	Unverified	1956	USDA	Africa	1
PI 241589	Unverified	1957	USDA	Asia	3
PI 241609	Unverified	1957	USDA	Australia	1
PI 241611	Unverified	1957	USDA	Australia	5
PI 241612	Unverified	1957	USDA	Australia	1

Accession	Type	Year of collection	Genebank	Origin	DAPC_cluster
PI 241617	Unverified	1957	USDA	Australia	3
PI 241620	Unverified	1957	USDA	Australia	3
PI 244663	Unverified	1957	USDA	Asia	3
PI 244830	Unverified	1958	USDA	North America	5
PI 244831	Unverified	1958	USDA	North America	5
PI 244833	Unverified	1958	USDA	North America	5
PI 244834	Unverified	1958	USDA	North America	1
PI 249533	Commercial vegetable seeds	1958	USDA	Europ	1
PI 250127	Unverified	1958	USDA	Asia	5
PI 261598	Cultivar	1959	USDA	North America	1
PI 261599	Unverified	1959	USDA	North America	1
PI 264651	Unverified	1960	USDA	Europ	2
PI 264658	Unverified	1960	USDA	Europ	3
PI 267722	Unverified	1960	USDA	North America	1
PI 267724	Unverified	1960	USDA	North America	5
PI 269311	Unverified	1960	USDA	Europ	5
PI 269312	Unverified	1960	USDA	Europ	3
PI 269313	Unverified	1960	USDA	Europ	4
PI 269315	Unverified	1960	USDA	Europ	4
PI 271320	Unverified	1960	USDA	Asia	3
PI 271445	Collector	1961	USDA	Asia	5
PI 274783	Unverified	1961	USDA	Asia	3
PI 277272	Unverified	1961	USDA	Asia	3
PI 277273	Unverified	1961	USDA	Asia	3
PI 277275	Unverified	1961	USDA	Asia	5
PI 284594	Unverified	1962	USDA	Europ	4
PI 285061	Unverified	1962	USDA	Europ	4
PI 285062	Unverified	1962	USDA	Europ	4
PI 285275	Unverified	1962	USDA	Europ	3
PI 289693	Unverified	1963	USDA	Australia	1
PI 289694	Unverified	1963	USDA	Australia	1
PI 289695	Unverified	1963	USDA	Australia	1
PI 289696	Unverified	1963	USDA	Australia	1
PI 291993	Unverified	1963	USDA	Asia	5
PI 291995	Unverified	1963	USDA	Asia	3
PI 291996	Unverified	1963	USDA	Asia	4
PI 296130	Unverified	1964	USDA	North America	5
PI 320997	Unverified	1967	USDA	Asia	5
PI 320998	Unverified	1967	USDA	Asia	3
PI 320999	Unverified	1967	USDA	Asia	3
PI 321001	Unverified	1967	USDA	Asia	3
PI 343474	Unverified	1969	USDA	Europe	5

Accession	Type	Year of collection	Genebank	Origin	DAPC_cluster
PI 343475	Unverified	1969	USDA	Europe	3
PI 343476	Unverified	1969	USDA	Europe	4
PI 343478	Unverified	1969	USDA	Europe	3
PI 343479	Unverified	1969	USDA	Europe	5
PI 343481	Unverified	1969	USDA	Europe	2
PI 343482	Unverified	1969	USDA	Europe	3
PI 344269	Unverified	1969	USDA	Asia	1
PI 462216	Unverified	1981	USDA	Europ	1
PI 462223	Unverified	1981	USDA	Europ	5
PI 462225	Unverified	1981	USDA	Europ	5
BRA 1334	Cultivar	1965	IPK	Europ	2
BRA 1335	Cultivar	1971	IPK	Europ	2
BRA 1336	Cultivar	1971	IPK	Europ	4
BRA 1338	Cultivar	1970	IPK	Europ	2
BRA 1339	Cultivar	1971	IPK	Europ	2
BRA 1340	Cultivar	1971	IPK	Europ	2
BRA 1341	Cultivar	1970	IPK	Europ	2
BRA 1342	Cultivar	1971	IPK	Europ	2
BRA 1343	Cultivar	1970	IPK	Europ	2
BRA 1344	Cultivar	1970	IPK	Europ	2
BRA 1345	Cultivar	1971	IPK	Europe	4
BRA 1346	Cultivar	1971	IPK	Europ	2
BRA 1347	Cultivar	1971	IPK	Europ	2
BRA 1348	Cultivar	1970	IPK	Europ	2
BRA 1349	Breeding material	1970	IPK	Europ	2
BRA 1351	Cultivar	1971	IPK	Europ	4
BRA 1352	Cultivar	1971	IPK	Europ	4
BRA 1353	Cultivar	1970	IPK	Europ	2
BRA 1354	Cultivar	1971	IPK	Europ	2
BRA 1355	Cultivar	1971	IPK	Europ	2
BRA 1357	Cultivar	1971	IPK	Europ	2
BRA 1359	Cultivar	1970	IPK	Europ	4
BRA 1363	Cultivar	1971	IPK	Europe	4
BRA 1364	Cultivar	1970	IPK	Europ	4
BRA 1365	Cultivar	1971	IPK	Europ	4
BRA 1366	Cultivar	1970	IPK	Europe	4
BRA 1367	Cultivar	1971	IPK	Europ	4
BRA 1368	Breeding material	1971	IPK	Europ	2
BRA 1369	Cultivar	1971	IPK	Europ	2
BRA 1370	Cultivar	1971	IPK	Europ	2
BRA 1371	Cultivar	1971	IPK	Europ	4
BRA 1372	Cultivar	1971	IPK	Europ	2

Accession	Type	Year of collection	Genebank	Origin	DAPC_cluster
BRA 1373	Cultivar	1969	IPK	Europ	4
BRA 1374	Cultivar	1969	IPK	unknown	2
BRA 1375	Cultivar	1971	IPK	unknown	4
BRA 1376	Cultivar	1971	IPK	unknown	4
BRA 1377	Cultivar	1969	IPK	Europ	2
BRA 1379	Cultivar	1972	IPK	unknown	2
BRA 1380	Cultivar	1972	IPK	Europ	4
BRA 1381	Cultivar	1971	IPK	unknown	2
BRA 1383	Cultivar	1971	IPK	unknown	2
BRA 1384	Cultivar	1971	IPK	unknown	2
BRA 1385	Cultivar	1974	IPK	Europ	2
BRA 1386	Cultivar	1974	IPK	Europ	2
BRA 1387	Cultivar	1974	IPK	Europ	2
BRA 1388	Cultivar	1974	IPK	Europ	2
BRA 1389	Cultivar	1972	IPK	Europ	2
BRA 1391	Cultivar	1974	IPK	Europ	2
BRA 1392	Cultivar	1972	IPK	Europ	2
BRA 1393	Cultivar	1969	IPK	Europ	2
BRA 1394	Cultivar	1972	IPK	Europ	2
BRA 1397	Cultivar	1974	IPK	Europ	4
BRA 1398	Cultivar	1974	IPK	Europ	2
BRA 1399	Cultivar	1974	IPK	Europ	2
BRA 1400	Breeding material	1974	IPK	Europ	2
BRA 1401	Cultivar	1974	IPK	Europ	2
BRA 1402	Cultivar	1974	IPK	Europ	2
BRA 1403	Cultivar	1974	IPK	Europe	2
BRA 1406	Cultivar	1974	IPK	Europe	4
BRA 1407	Breeding material	1974	IPK	Europe	4
BRA 1408	Cultivar	1974	IPK	Europ	5
BRA 1409	Cultivar	1974	IPK	Europe	4
BRA 1410	Cultivar	1986	IPK	unknown	2
BRA 1411	Landrace	1987	IPK	Asia	1
BRA 1414	Cultivar	1990	IPK	Europ	2
BRA 1415	Cultivar	1971	IPK	Australia	1
BRA 1416	Landrace	1981	IPK	Europ	4
BRA 1480	Cultivar	1971	IPK	Europe	4
BRA 1481	Cultivar	1972	IPK	Europe	4
BRA 1482	Cultivar	1974	IPK	Europ	4
BRA 1483	Cultivar	1974	IPK	unknown	4
BRA 163	Landrace	1957	IPK	Asia	4
BRA 1750	Hybrid	1994	IPK	Europ	3
BRA 1785	Cultivar	2001	IPK	Europ	2

Accession	Type	Year of collection	Genebank	Origin	DAPC_cluster
BRA 1789	Landrace	1991	IPK	Europ	1
BRA 2039	Cultivar	2002	IPK	Europ	2
BRA 2057	Cultivar	2002	IPK	Europ	2
BRA 2058	Cultivar	2002	IPK	Europ	2
BRA 290	Cultivar	1965	IPK	Europ	2
BRA 283	Cultivar	1963	IPK	Europ	2
BRA 284	Cultivar	1963	IPK	Europ	4

Table S2 Summary of phenotypic variation and estimates of broad-sense heritability (H^2) for all traits. Influence of Genotype (G), Environment (E), and Genotype-Environment interaction (G×E) were tested using an ANOVA. *** Significant at $p < 0.001$.

	Trait value					
Trait	Mean±SD	Range	G	E	G×E	H^2
Curd width	13.52±0.73	11.11-15.29	***	***	***	0.437
Cluster width	4.38±0.39	2.86-5.28	***	***	***	0.564
No. of branches	11.17±0.59	9.71-13.40	***	***	***	0.264
Apical length	1.19±0.14	0.74-1.65	***	***	***	0.111
Nearest branch	1.68±0.17	1.19-2.23	***	***	***	0.050
No. of days	75.23±11.68	45.57-118.43	***	***	***	0.943

Table S3 Phenotypic and genotypic correlation coefficients between traits. Values below the diagonal represent phenotypic correlation coefficients (correlation of BLUEs). Values above the diagonal are genetic correlation coefficients (correlation of BLUPs). ** Significant at $P < 0.01$, *** Significant at $P < 0.001$.

Trait	Curd width	Cluster width	No. of branches	Apical length	Nearest branch	No. of days
Curd width	-	0.59	0.004	0.27	0.24	0.04
Cluster width	0.69***	-	0.07	0.25	0.32	-0.21
No. of branches	-0.27***	-0.16*	-	-0.27	-0.21	-0.23
Apical length	0.64***	0.42***	-0.41***	-	0.71	-0.03
Nearest branch	0.50***	0.50***	-0.37***	0.79***	-	-0.07
No. of days	0.09	-0.13	-0.22**	0.01	0.11	-

Table S4 Geographic origin according to USDA and IPK passport information of accessions for each inferred DAPC cluster.

Cluster	Africa	Asia	Australia	Europe	USA	Russia	Unknown origin	Cluster size
1	2	3	6	11	4	0	0	25
2	0	0	0	54	0	2	6	56
3	1	11	2	7	0	3	0	24
4	0	3	0	25	0	8	4	40
5	0	8	1	6	5	2	0	22

Table S5 Overview of λ values. Values close to one indicate no confounding due to population structure.

	Unimputed data		Imputed data	
	EMMAX	MLMM	EMMAX	MLMM
Curd width	1	0.87	1	0.98
Cluster width	1.08	0.98	1.02	1.02
No. of branches	1.09	1.13	0.995	1
Apical length	1.03	1.05	1.02	0.93
Nearest branch length	1.11	0.99	1.04	1.02
No. of days	1.18	1.18	1.01	1

Table S6 Phenotypic variance explained due to population structure. PC = Principle component.

PC	Curd width	Cluster width	No. branches	Length nearest	Apical lengt1	No. days
PC1	16.37	40.01	2.54	6.7	4.01	1.86
PC2	1.64	2.23	4.26	2.22	0.94	27.66
PC3	0.51	0	2.23	0.58	0.06	3.11

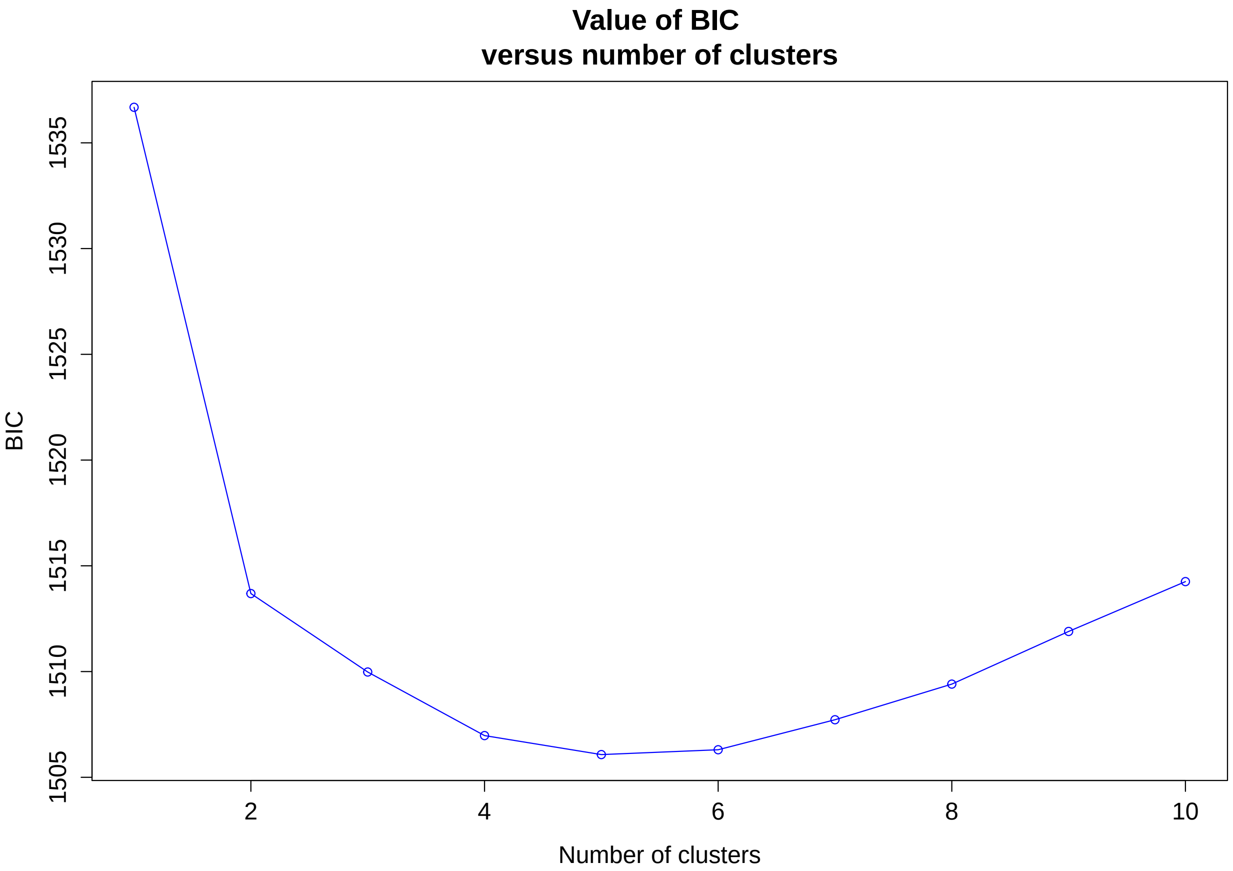

Figure S1 BIC values of the k-means clustering algorithm. Lowest value shows most likely number of clusters (5).

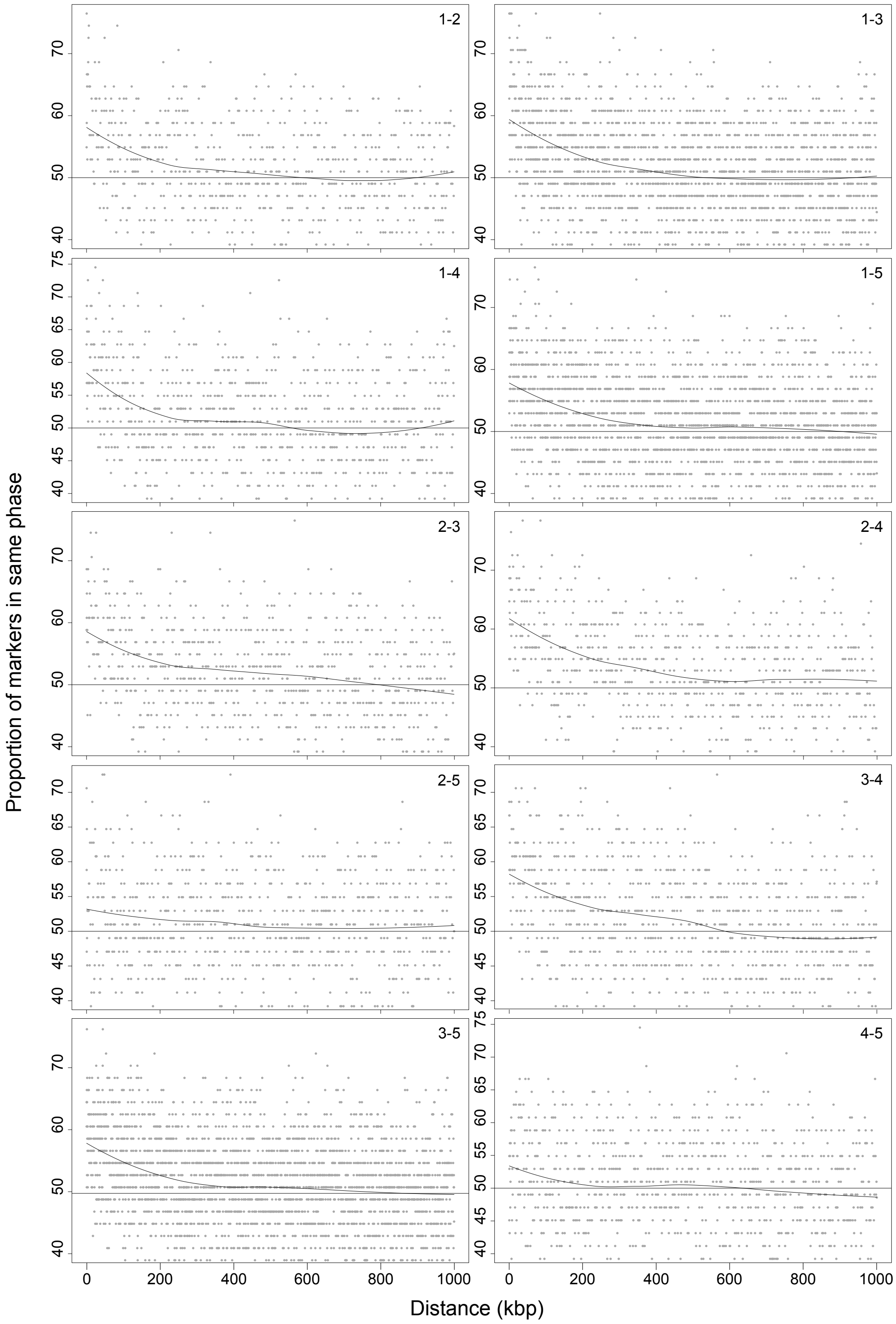

Figure S2 Persitence of linkage phase calculated based on the proportion of markers that share the same sign between same chromsomes in different cluster. Positions are the averages of groups of 50 markers.

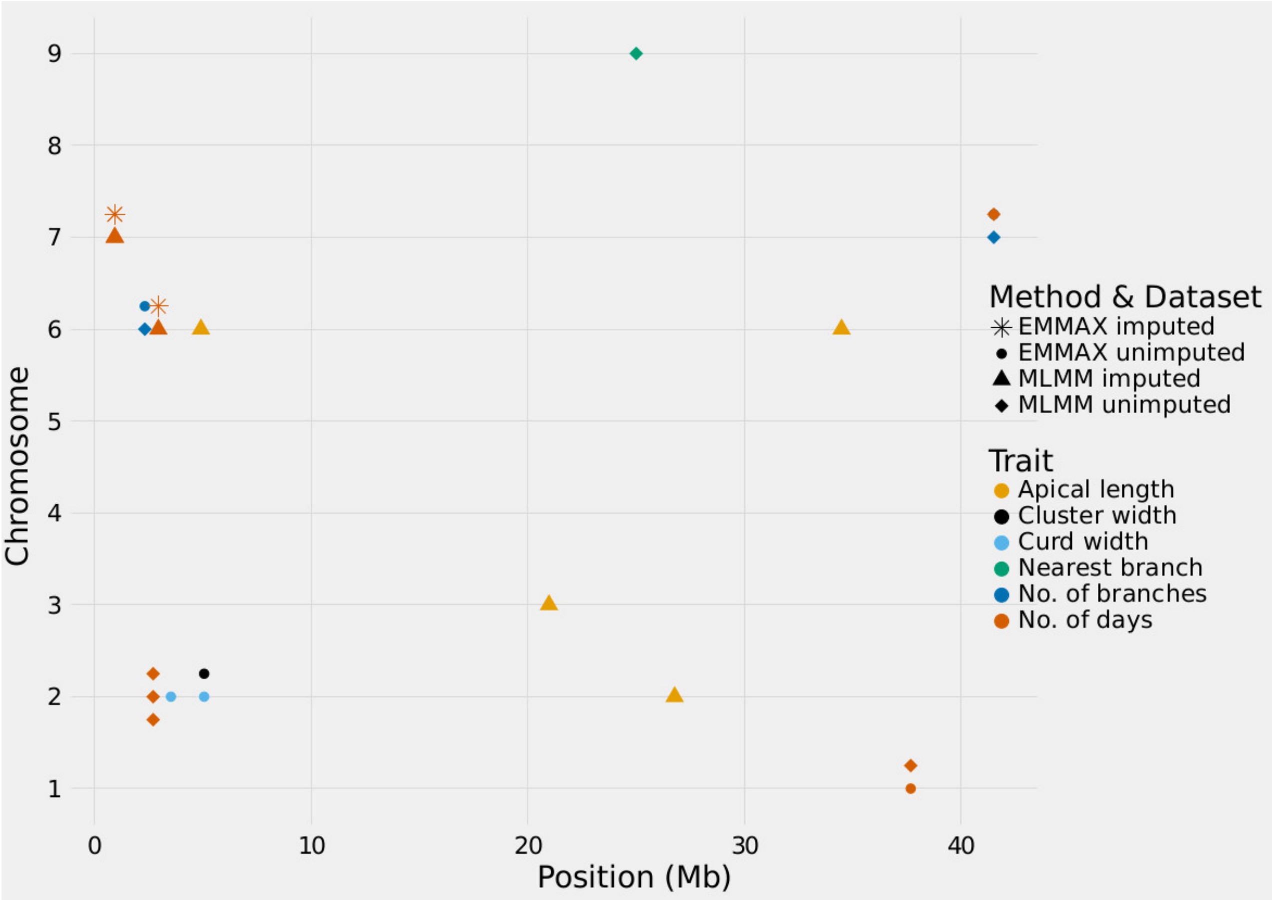

Figure S3 Overview, of the GWAS results for EMMAX and MLMM with the unimputed and imputed data sets for each trait, respectively.

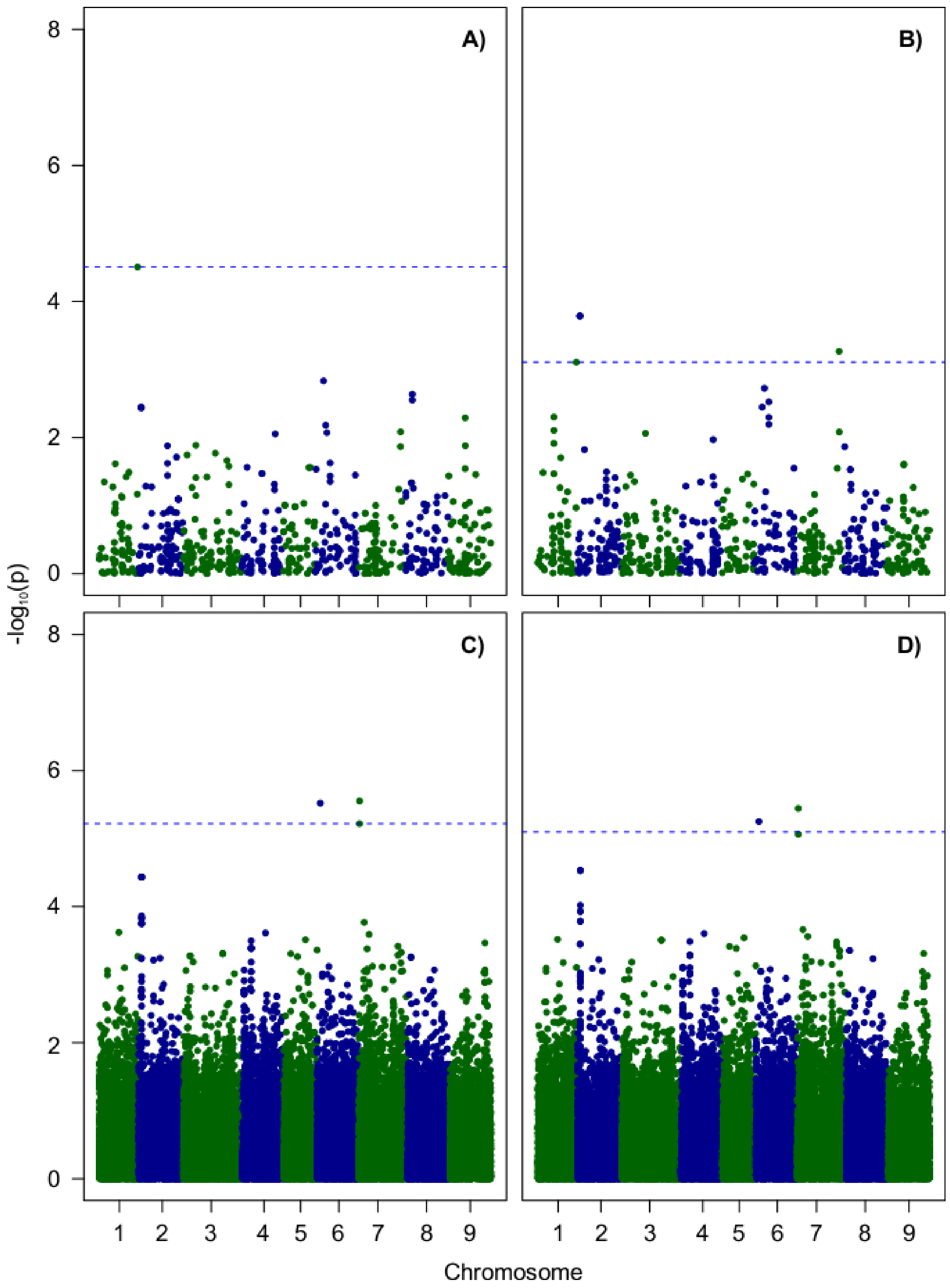

Figure S4 Manhattan plots of the GWAS results for the trait number of days to budding. a) EMMAX and b) MLMM results for the unimputed data set and c) EMMAX and d) MLMM result for the imputed data set. The blue dotted line is the significant threshold based on the FDR.

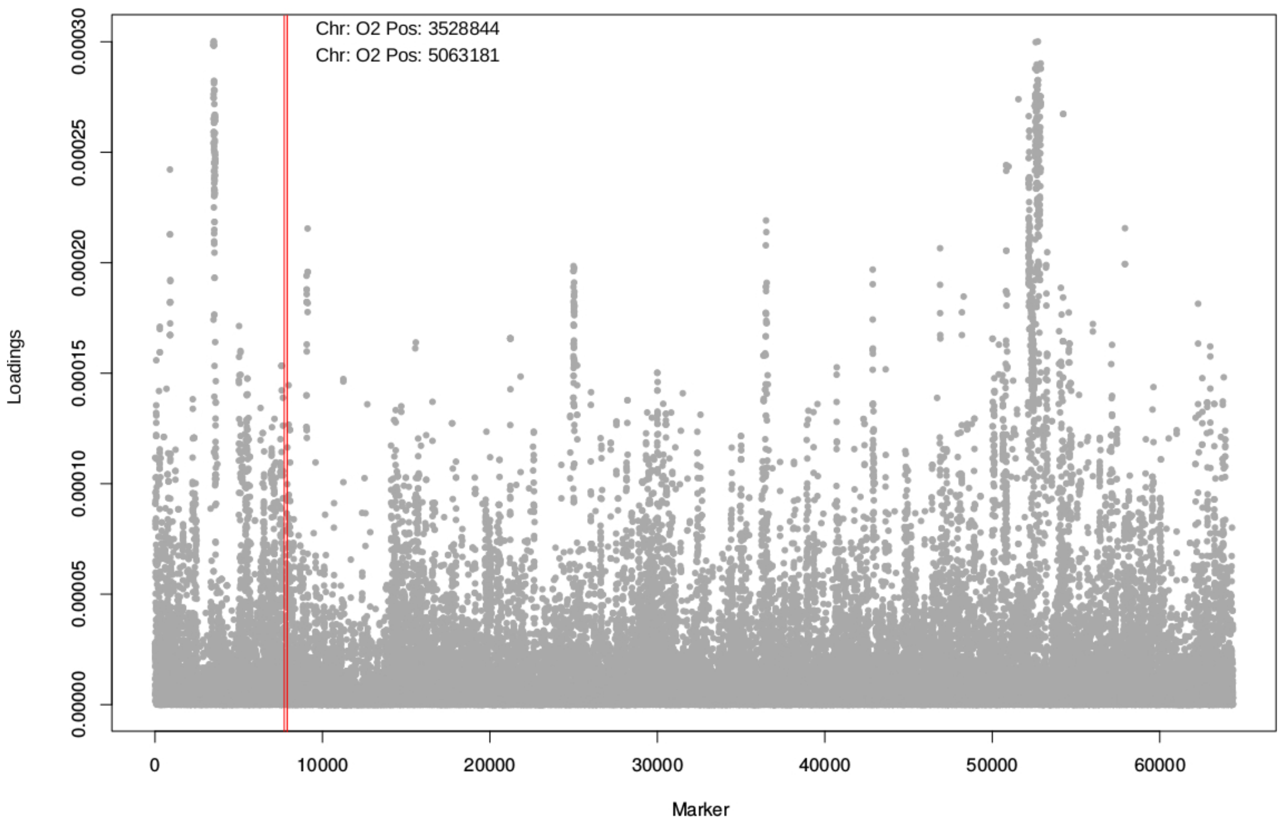

Figure S5 Loading plot of the first principle component using the imputed data set. Red lines show positions of significant QTLs detected for the traits curd width and cluster width using the unimputed data set.

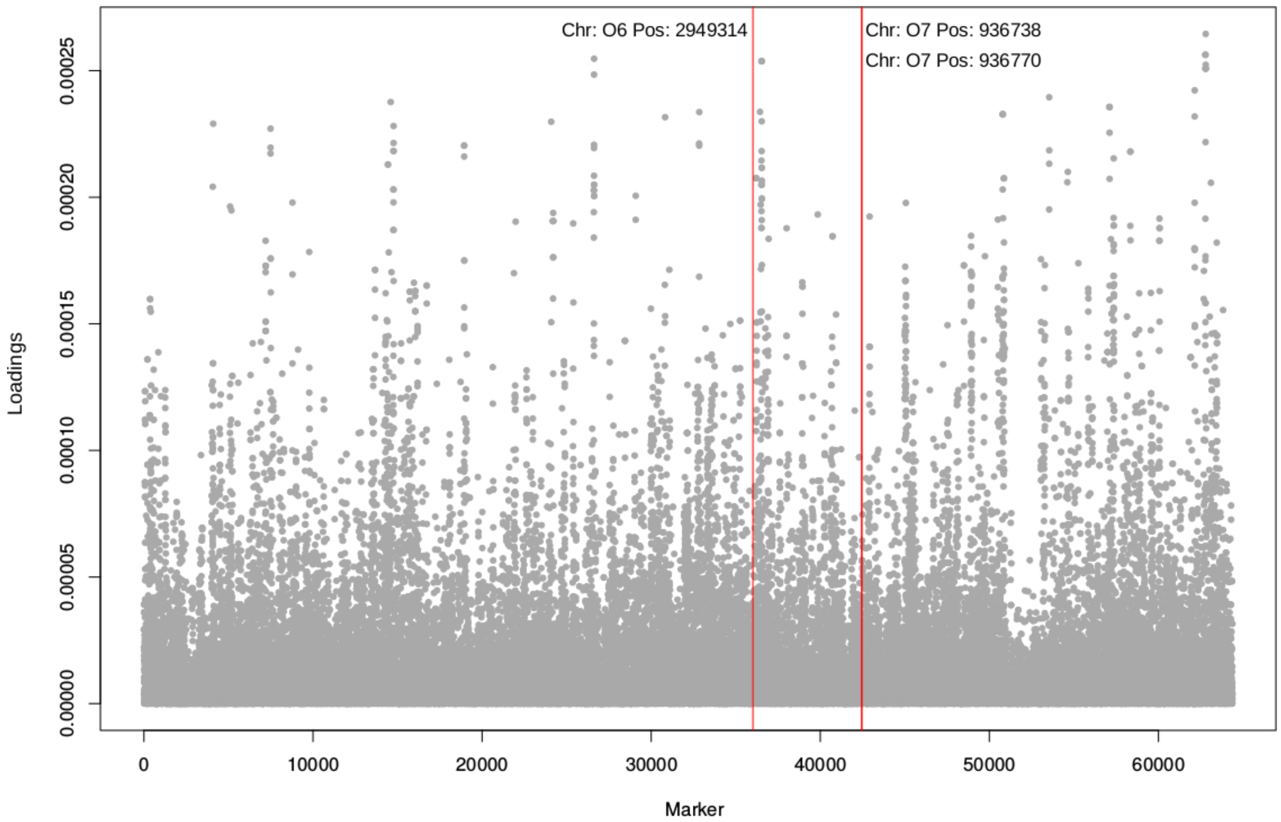

Figure S6 Loading plot of the second principle component using the imputed data set. Red lines show positions of significant QTLs detected for the trait no. of days to budding using the imputed data set.
